# A novel cuproptosis pattern and tumor immune microenvironment characterization in urothelial carcinoma of the bladder

**DOI:** 10.3389/fimmu.2023.1219209

**Published:** 2023-08-17

**Authors:** Huan Feng, Zhiyao Deng, Yibao Huang, Zhuo Liu, Yajun Ruan, Tao Wang, Jihong Liu

**Affiliations:** ^1^ Department of Urology, Tongji Hospital, Tongji Medical College, Huazhong University of Science and Technology, Wuhan, Hubei, China; ^2^ Institute of Urology, Tongji Hospital, Tongji Medical College, Huazhong University of Science and Technology, Wuhan, Hubei, China; ^3^ Shenzhen Huazhong University of Science and Technology Research Institute, Shenzhen, Guangdong, China; ^4^ Department of Thoracic Surgery, Tongji Hospital, Tongji Medical College, Huazhong University of Science and Technology, Wuhan, Hubei, China; ^5^ Department of Gynaecology, The Second Affiliated Hospital of Guangxi Medical University, Nanning, Guangxi, China

**Keywords:** cuproptosis, tumor immune microenvironment, urothelial carcinoma of the bladder, immunotherapy, prognosis

## Abstract

**Background:**

Urothelial carcinoma of the bladder (UCB) is the most prevalent malignant tumor of the urinary system worldwide, which has a significant recurrence rate despite multiple treatment options available. As a unique and novel copper-dependent programmed cell death mechanism, the comprehensive impact of cuproptosis on the tumor immune microenvironment, clinicopathological characteristics and the prognosis of patients remains largely unclear.

**Methods:**

A total of 568 UCB samples were thoroughly examined for cuproptosis patterns using data downloaded from TCGA and GEO, based on 10 cuproptosis-related genes reported previously. Then, the univariate COX regression analysis was performed on the genes that differed across the various patterns. To measure individual cuproptosis pattern, a cuproptosis score system was constructed using a principal component analysis algorithm. To validate the scoring system, immunohistochemical staining was performed on tumor tissues with different pathological grades, and experiments *in vitro* were conducted about the differentially expressed genes related to prognosis. Finally, the capacity of scoring system to predict the response to immunotherapy was verified by using data from IMvigor 210 cohort.

**Results:**

Four unique cuproptosis clusters and two gene clusters were finally found by the investigation. The clinical features and prognosis of patients, as well as the mRNA transcriptome, pathway enrichment, and immune cell infiltration in TME, varied dramatically between various cuproptosis clusters and gene clusters. To identify individual cuproptosis patterns in UCB patients, we also established a cuproptosis scoring system. After validation with multiple methods, it was indicated that the score system could predict the prognosis of UCB patients and was significantly connected to clinical features such TNM category, tumor grade, molecular type and ultimate survival status. The clinical outcomes of UCB patients were predicted effectively according to the tumor mutation burden in conjunction with the scoring system. Furthermore, we found that the cuproptosis score had a significant correlation with the response to immunotherapy and the sensitivity to chemotherapy.

**Conclusion:**

This study revealed the potential impact of cuproptosis on the UCB tumor immune microenvironment and clinical pathological characteristics. The cuproptosis score system could effectively predict the prognosis of patients and the response to chemotherapy and immunotherapy.

## 1 Introduction

Urothelial carcinoma of the bladder (UCB) is the most prevalent genitourinary system cancer, with high recurrence and mortality rates (1). The incidence of UCB ranks ninth among all malignant tumors, while its mortality ranks fourteenth (2). In 2020, a total of 573,278 new cases and 212,536 related deaths were reported worldwide (3). Although approximately 70-80% of UCB patients are initially diagnosed with non-muscle invasive bladder cancer (NMIBC), at least half of the patients will experience a recurrence within 5 years, and 10-30% of them will also develop muscle invasive bladder cancer (MIBC), which has worse prognosis and lower 5-year survival rate (4, 5). For NMIBC patients, the primary treatments are transurethral resection of the bladder (TURB) and intravesical chemotherapy or immunotherapy. For MIBC patients, radical cystectomy and lymphadenectomy may be operated according to the specific circumstances, combined with preoperative or postoperative radiotherapy, chemotherapy and neoadjuvant immunotherapy (6). Various researches have demonstrated the tumor microenvironment (TME) and genetic alterations occurring in cancer cells were directly associated with tumor progression and recurrence (7). Many patients have benefited from immune checkpoint inhibitors (ICI) targeting programmed cell death protein 1 (PD-1) and its ligand programmed cell death protein 1 (PD-L1), and these therapies are anticipated to prolong the survival time of MIBC patients (8). However, less than 20% of patients obtained an objective response to immunotherapy, because of the complexity of TME and individual variability (9). Therefore, it is urgently necessary to open up novel perspectives and strategies due to current treatments are insufficient to satisfy the needs of patients and clinical therapy.

Regulated cell death (RCD), defined as an active and orderly process of cell death regulated genetically, resulting in unique biochemical, functional and immunological effects through precise signaling cascades and defined effector molecules (10). This phenomenon is widespread in the development of organisms and can be classified into more than 20 different categories based on various molecular mechanisms and biological consequences, among which apoptosis, necroptosis, pyroptosis and ferroptosis are considered to be closely associated with tumorigenesis (11, 12). Apoptosis is the most intensively researched type and a major contributor to cancer (13). The effects of multiple clinical treatments, including chemotherapy, targeted therapy and immunotherapy, frequently depend on the sensitivity of cancer cells to apoptosis (14, 15). Necrosis can serve as a protective mechanism to prevent the progression of cancer (16), but it may also promote carcinogenesis by generating adaptive immunity (17). Ferroptosis is a signal that enhances the generation of lipid reactive oxygen species (ROS) in many tumors and causes a catastrophic accumulation of lipid peroxides on the cell membrane, which results in cell death (18). Ferroptosis has been reported to be a valuable therapeutic target for highly aggressive and drug-resistant cancers, such as pancreatic ductal adenocarcinoma (PDAC) (19). Recently, *Science* reported a unique RCD mechanism—cuproptosis, which is characterized by copper-induced cell death, and its particular mitochondrial metabolic mechanism is expected to provide a novel target for tumor therapy (20).

Copper is a crucial trace element for human body, to function properly in the neurological system and hematological system, as well as to regulate metabolism and immunity and to resist oxidative damage. Lack of copper ions affects the function of copper-binding enzymes and impairs physiological homeostasis, while an excess of copper can cause mitochondrial protein aggregation and various forms of cell death (21). Cuproptosis is the process in which a significant number of copper ions directly attach to the lipoylated protein components of the tricarboxylic acid cycle (TAC), leading to the aggregation of lipoylated proteins and the loss of Fe-S cluster proteins, which in turn causes proteotoxic stress and cell death (22). It is characterized by a dependence on the production of adenosine triphosphate (ATP) through mitochondrial respiration rather than glycolysis (20, 23). Existing evidence indicated that an imbalance in copper is tightly related to cancers like breast cancer, colorectal cancer and prostate cancer (24, 25). However, copper contributes in biological processes such as tumor cell proliferation, invasion, angiogenesis and metastasis through a variety of molecular pathways (26, 27). In the respiratory state, tumor cells may express more lipoylated protein, leading to an increase in protein aggregates. Additionally, the metabolic flux of lipoylated protein rises, and their affinity for copper is enhanced, both of which cause more protein aggregation again (21). There might be a window period in tumors when the concentration of copper ions increased, allowing the cancer cells to be killed off specifically (28). Cuproptosis frequently eliminates cancer cells preferentially compared to normal cells, thereby improving the selectivity of treatment, decreasing tumor resistance, and minimizing adverse effects (29). Cuproptosis serves as not only a target for cancer therapy, but also the foundation for estimating risk and prognosis of cancer, which has considerable research significance.

In this study, using 568 samples from the TCGA-BLCA cohort and GEO datebase, we assessed the genetic variation of 10 cuproptosis-regulated genes (CRGs) in UCB and categorized the patients into various cuproptosis patterns based on the expression of CRGs. Next, we predicted the characteristics of immune cell infiltration in TME and discovered that cuproptosis patterns had an intense connection with TME. Then, we identified 150 cuproptosis cluster-related differentially expressed genes (DEGs). The patients were divided into different gene-subgroups, and investigated the association between cuproptosis patterns and gene patterns. Moreover, we constructed a scoring system to measure the cuproptosis pattern of individual patient, and investigated the features of cuproptosis in tumor mutation burden, immunotherapy and chemotherapy. Finally, in order to verify the scoring system, DEGs related to prognosis were validated by immunohistochemical staining and experiments *in vitro.*

## 2 Method

### 2.1 Data acquisition and preprocessing

RNA-sequencing expression profiles and clinical information of UCB patients were downloaded from the public databases Cancer Genome Atlas (TCGA database, https://portal.gdc.cancer.gov/) and Gene Expression Omnibus (GEO database, www.ncbi.nlm.nih.gov/geo). To minimize statistical errors, we excluded cases of incomplete clinical data. After screening, TCGA-BLCA cohort and GSE13507 dataset were included in the research. To facilitate differential analysis, we convert the fragments per kilobase of transcript per million mapped reads (FPKM) values to transcripts per kilobase million (TPM) values in TCGA-BLCA cohort. The mutation atlas was also derived from the TCGA database. The copy number variation (CNV) matrix was acquired from UCSC-Xena (http://xena.ucsc.edu/). We used the “Combat” algorithm to remove the batch effects between TCGA-BLCA cohort and GSE13507 dataset by R package “sva”, and merged them for subsequent analysis. In addition, we downloaded RNA sequencing expression profiles and sample clinical information from the GSE19915 dataset as an independent validation set.

### 2.2 Consensus clustering analysis based on CRGs

A total of 10 CRGs were retrieved from the study of Tsvetkov et al. (20). Based on the expression profiles of 10 CRGs, unsupervised consensus clustering analysis was performed using “Consensus Cluster Plus” in the R package, and the patients were divided into various clusters. To guarantee and confirm the stability of supervised clustering, all procedures were repeated 1000 times. A combination of cumulative distribution function (CDF) curves, scree plot and track plot were considered to determine the most appropriate number of clusters.

### 2.3 Assessment of the cell infiltration status in TME

To clarify the survival differences between various clusters, the Kaplan-Meier curve was plotted for the cuproptosis clusters. We investigated the differentiation between several cuproptosis clusters using principal component analysis (PCA). Besides, the gene set variation analysis (GSVA) was performed with “GSVA” in the R package to detect the different biological functions between distinctive clusters. The hallmark gene sets utilized in GSVA were derived from the MSigDB database. Three tools were utilized to assess the relative abundance of immune cell infiltration and immune functions among various clusters: single-sample gene set enrichment analysis (ssGSEA), CIBERSORT, and XCELL. The ESTIMATE algorithm was applied to calculate the immune score, stromal score and tumor purity for every cluster.

### 2.4 Generation of DEGs among cuproptosis clusters, functional annotation and enrichment analysis

To identify DEGs, differential expression analysis was carried out between every two cuproptosis clusters using “limma” in the R package. The final DEGs were generated from the intersection of these differently compared DEGs. The Gene Ontology (GO) and Kyoto Encyclopedia of Genes and Genomes (KEGG) functional enrichment analysis of DEGs were performed with “cluster Profiler” in the R package to explorer the possible functions and mechanisms of DEGs. GO and KEGG gene sets were downloaded from MSigDB database also. Univariate COX regression was applied to identify DEGs with prognostic value. After that, we conducted unsupervised consensus clustering analysis once more and identified distinctive gene-subgroups. Then, we performed GSVA analysis and GO enrichment between the various subgroups.

### 2.5 Establishment of the cuproptosis scoring system (CSS) model

Furthermore, PCA was performed to differentiate the molecular signature patterns of the DEGs with prognostic value, and the cuproptosis score formula was established:


CSS=∑(PC1i+PC2i)

In the formula above, PC1i and PC2i respectively represent the two-dimensional expression scores of DEGs, and the total of two scores is cuproptosis score, which reflects the cuproptosis pattern of each individual to a certain extent.

We defined the threshold based on median risk score (cut-off value=0.05) in the training set, and patients were divided into high CSS score group and low CSS score group. Using “survminer” from the R package, the survival analysis was carried out to determine the connection between the cuproptosis score and survival. Additionally, we utilized univariate and multivariate Cox regression to test whether the CSS model was a reliable predictor of prognosis, and identify variables that independently characterized prognostic risk. GSE19915 served as an independent dataset for external validation of the CSS model.

### 2.6 Immunohistochemistry

To validate the CSS model at clinical level, immunohistochemical (IHC) staining was utilized to detect the expression of these prognostic DEGs in UCB tissues with different pathological grades. The research acquired the approval from the Institutional Review Committee of Tongji Hospital, Tongji Medical College, Huazhong University of Science and Technology (TJ-IRB20221316). We collected the UCB tissues with various pathological grades from Tongji Hospital after obtaining the written informed consent of patients. Then, IHC staining was operated using the vecastain EliteABC kit (Vector Laboratories, Burlingame, CA, USA), and its procedures were described as previously described (30). Primary antibodies against P4HB (Cat No. A19239) and Calreticulin (Cat No. A20986) were purchased from ABclonal (Wuhan, China). Primary antibodies against PRDX1 (Cat No.15816-1-AP), EIF1 (Cat No.15276-1-AP) and TXNIP (Cat No.18243-1-AP) were purchased from Proteintech (USA). Image Pro Plus software was used to analyze and quantify the IHC results. All experiments were carried out independently at least three times, and the typicality of the selected tissues were ensured by the pathologist.

### 2.7 Cell culture, lentivirus plasmid construction and transfection

Human bladder cancer cell lines T24 and 5637 were cultured in 1640 medium (Boster, Wuhan, China) supplemented with 10% fetal bovine serum (FBS, Gibco, CA, USA). Human embryonic kidney cell line 293T was cultured in DMEM medium (Boster, Wuhan, China) supplemented with 5% FBS (Gibco, CA, USA) and 1% penicillin/streptomycin. Cells were incubated at 37°C with 5% CO_2_. The sh-RNA sequences corresponding to P4HB, PRDX1 and Calreticulin were designed using online tools (https://www.sigmaaldrich.cn) and synthesized by Qingke Biotechnology Co., LTD (Beijing, China). Then, the target sequences were inserted into the pLKO.1 vector through restriction sites at both ends and transformed into DH5α E. coli competent cells (Qingke, Beijing, China). Plasmids were extracted using an endotoxin free plasmid kit (Zoman, Beijing, China), and the three-plasmid psPAX2, pMD2.G and target plasmid co-transfection system was constructed. Next, the supernatant of 239 T cell was collected at 48 hours after transfection. Filtering the media through a 0.45 μm filter to remove cells, the supernatant was concentrated by lentivirus concentration solution (Yeasen, Shanghai, China). Finally, T24 and 5637 cells in logarithmic growth phase were transfected by the concentrated lentivirus, which were continued in culture. After 72 h of transfection, the cell knockdown efficiencies were assessed by qRT-PCR. The detailed procedure of the above operation was displayed on this website (https://www.addgene.org).

### 2.8 Real-time quantitative PCR

Total RNA was extracted according to TRIzol reagent (Servicebio, Wuhan, China). cDNA was obtained by reverse transcription using the cDNA synthesis supermix kit (Yeasen, Shanghai, China). The 20 μL qRT-PCR system was established according to the qPCR SYBR Green master mix kit (Yeasen, Shanghai, China). β-actin was served as internal reference. The relative expression levels were calculated according to the 2^−△△Ct^ method. qPCR was performed with three independent experiments. *p*-values< 0.05 were regarded as statistically significant. The sh-RNA sequences and primers sequences (5’-3’) were showed in **Supplementary Table S1**.

### 2.9 Cell proliferation and migration assays

The sequence with the maximum knockdown efficiency was selected for cell proliferation and migration experiments. The stably transfected T24 and 5637 cells were divided into different groups and seeded onto a 96-well plate at a density of 1×10^3^ cells/well. After incubation of 24h, 48h and 72h respectively, the cell counting kit-8 (CCK-8, Yeasen, Shanghai, China) was mixed at 10μL/well and incubated at 37°C with 5% CO_2_ for 2 h. Finally, the optical density (OD) values at 450 nm were obtained on an automatic microplate reader (Thermo Fisher, USA). For colony formation assay, lentivirus-transfected T24 and 5637 cells were digested and resuspended (1×10^3^ cells/mL). The cell suspension (1 mL) was seeded onto 12-well plate and cultured for 14 days, the medium was changed every 3 days. Cells were fixed with 4% paraformaldehyde (Servicebio, Wuhan, China), stained with 0.1% crystal violet solution (Servicebio, Wuhan, China), and the number of colonies was counted by Image J software. The transwell assay was performed to detect the migration ability of cells. The stably transfected T24 and 5637 cells were collected and resuspended in serum-free medium (2×10^5^ cells/mL), and the cell suspension (100μL) was incubated in upper chamber. In addition, 700 μL of medium containing 15% FBS was added to the lower chamber. After incubated in an incubator for 18h, the cells were fixed with 4% paraformaldehyde (Servicebio, Wuhan, China), stained with 0.1% crystal violet solution (Servicebio, Wuhan, China), and the cells which migrating to the lower surface of the transwell chamber were counted under microscope. All experiments were repeated three times independently, and *p*-values< 0.05 were regarded as statistically significant.

### 2.10 Assessment of the mutational frequencies and cell infiltration characteristics in TME between different CSS score groups

Tumor mutational burden (TMB) refers to the number of mutations per megabase of DNA sequence in a specific tumor, obtained by calculating the somatic mutation profile of each sample. We evaluated the connection between CSS score and TMB, as well as combined the two variables to forecast survival in UCB patients. In order to compare the mutational frequencies of each gene between high and low CSS score groups, we used “maftools” in the R package to generate the mutational profiles of patients with high or low CSS scores respectively. We also utilized ssGSEA, CIBERSORT and XCELL to access relative abundance of immune cell infiltration in TME between the distinctive groups.

### 2.11 Chemotherapy and immunotherapy sensitivity assessment

To evaluate the response of UCB patients to chemotherapy medicines, the half-maximal inhibitory concentration (IC50) of chemotherapy drugs (cisplatin, gemcitabine, mitomycin C, methotrexate) was calculated by using “pRRophetic” in the R package. In order to predict immunotherapy response in patients with various CSS scores, two distinct approaches were applied: The Cancer Immunome Atlas (TCIA, https://tcia.at/home) (31) and Tumor Immune Dysfunction and Exclusion (TIDE) (32). The sensitivity to chemotherapy and immunotherapy were compared between groups with high and low CSS score by Wilcoxon test. Finally, we calculated the CSS scores for each patients in the IMvigor-210 cohort and compared the response to anti-PD-L1 immunotherapy between the high and low CSS score groups in order to validate the predictive efficacy of the CSS model to immunotherapy.

### 2.12 Statistical analysis

All raw data is processed and analyzed by R program version 4.1.2. The t-test was used to compare two groups of measurement data with normal distribution, while analysis of variance was used to compare more than two groups. If the normal distribution was not observed, the Wilcoxon rank-sum test and Kruskal-Wallis test were applied. For the purpose of comparing count data between groups, the Chi-square test was employed. The Kaplan-Meier method and log-rank test were utilized to establish survival curves and survival differences between the two groups respectively. The criteria of |log2-fold change (FC)| ≥ 1and *p*-value< 0.05 was selected for identification the DEGs and gene set enrichment analysis. *P*-values are on both sides, and *p*-values< 0.05 are considered statistical significance.

## 3 Result

### 3.1 Expression and mutation of CRGs in UCB

Ten critical genes involved in cuproptosis, defined as CRGs, were identified by Tsvetkov et al. through whole-genome CRISPR-Cas9 screening. Among them, seven genes conferred resistance to cuproptosis, including ferredoxin 1 (FDX1), lipoyl synthase (LIAS), lipolytransferase1 (LIPT1), dihydrolipoamide dehydrogenase (DLD), dihydrolipoamide S-acetyltransferase (DLAT), pyruvate dehydrogenase E1 subunit alpha 1 (PDHA1) and pyruvate dehydrogenase E1 subunit beta (PDHB). Meanwhile, there were three genes sensitized the cells to cuproptosis, including regulatory transcription factor 1 (MTF1), glutaminase (GLS), and cyclin dependent kinase inhibitor 2A (CDKN2A) (20).

**Figure 1:**
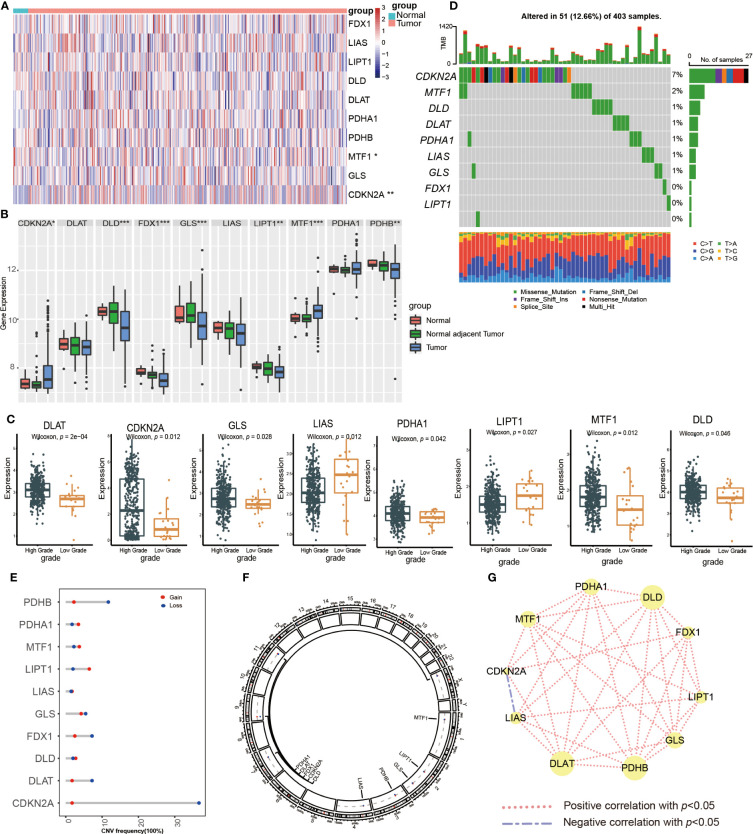
Transcriptional expression and mutation of cuproptosis-regulated genes (CRGs) in urothelial carcinoma of the bladder (UCB). **(A)** The expression of 10 CRGs between normal tissues and tumor tissues in the TCGA-BLCA cohort. **(B)** The expression of 10 CRGs between normal tissues, normal adjacent tumor tissues and tumor tissues in the GSE13507 dateset. **(C)** The expression of 10 CRGs between high and low grade tumor tissues in the TCGA-BLCA cohort. **(D)** Comprehensive analysis of somatic mutation frequency of 10 CRGs from the TCGA-BLCA cohort. **(E)** The copy number variation (CNV) frequency of CRGs in the TCGA-BLCA cohort. **(F)** The chromosomal location of CNV alterations in CRGs. **(G)** Spearman correlation of CRGs in UCB (*p< 0.05, **p< 0.01, ***p< 0.001).

First, we examined the expression of CRGs between normal bladder tissues and UCB tissues using TCGA-BLCA cohort. The results demonstrated that the expression of MTF1 was down-regulated while the level of CDKN2A was up-regulated in tumor tissues (**Figure 1A**, **Supplementary Figure S1A**). We also discovered 8 CRGs were expressed differently across various pathological categories. In the high-grade group, the levels of LIAS and LIPT1 were decreased, while the expression of six genes (DLAT, CDKN2A, GLS, PDHA1, MTF1, DLD) were increased (**Figure 1C**). After that, we analyzed the date of tumor tissues, normal adjacent tumor tissues and normal tissues from GSE13507 dataset. In tumor tissues, the abundance of CDKN2A and MTF1 were higher, as well as the levels of DLD, FDX1, GLS, LIPT1 and PDHB were down-regulated (**Figure 1B**). Overall, we found majority cuproptosis-facilitating genes were up-regulated in the high-grade tumor samples. However, the result was not absolute. We speculated this phenomenon was caused by high degree of heterogeneity in the inheritance and expression of CRGs between normal tissues and tumor tissues, and the mutation analysis was required to clarify the reason.

Next, we analyzed the somatic mutation and CNV of 10 CRGs in UCB samples. For 403 samples from TCGA-BLCA cohort, 51 samples had CRGs mutations with a frequency of 12.66%. CDKN2A had the highest mutation frequency of 7%, followed by MTF1, while FDX1, LIPT1 and PDHB showed no mutation (**Figure 1D**). The analysis of CNV revealed copy number loss was the most frequent CNV alteration. Among them, CDKN2A, PDHB, DLAT and FDX1 were more frequently expressed as copy number losses, while PDHA1, MTF1 and LIPT1 were predominantly expressed as copy number gains (**Figure 1E**). **Figure 1F** showed the location of CRGs on chromosomes with CNV alterations. These findings demonstrated that genes with a high frequency of somatic mutations, such as CDKN2A and MTF1, were up-regulated in the high-grade tumor samples. Although somatic mutations and CNV exhibited the critical role in regulating the expression pattern of CRGs, the mutational frequency was not completely consistent with all mRNA expression alterations. It suggested that there might be additional mechanisms of regulation, such as epigenetic regulation, RNA splicing or transcription factors. The results also revealed the dysregulation of CRGs and heterogeneity of genetic alterations could play a significant role in the occurrence and development of UCB. Finally, we performed the Spearman correlation analysis between CRGs, and found that CRGs showed significant and positive correlations with each other (**Figure 1G**).

### 3.2 Identification of cuproptosis subtypes based on CRGs

**Figure 2:**
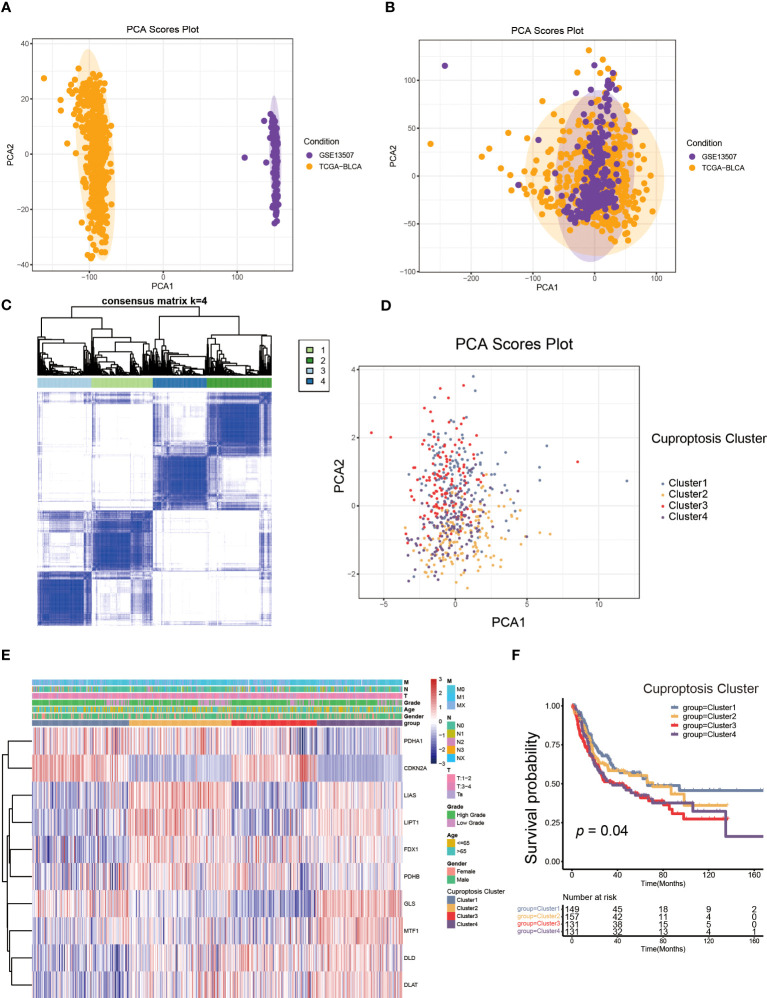
The generation of cuproptosis subtypes and clinicopathological characteristics of four subtypes. **(A, B)** Principal component analysis (PCA) for the expression profiles of common genes with the combination of the TCGA-BLCA cohort and the GSE13507dateset. **(C)** The unsupervised consensus clustering analysis of the training set for k=4. **(D)** PCA revealing the significant difference in transcriptomes of four subtypes. **(E)** The differences in the expression of CRGs and clinicopathological characteristics among four distinct cuproptosis clusters. **(F)** The differences in overall survival (OS) among four cuproptosis clusters by Kaplan-Meier curves.

To better elucidate the biological value and clinical significance of CRGs in UCB, we categorized UCB patients according to the expression of 10 CRGs. Before this, we utilized PCA to distinguish the data from TCGA-BLCA cohort and GEO dataset GSE13507 (**Figure 2A**). Then, we eliminated heterogeneity between the two datasets, and successfully combined them after consistent clustering (**Figure 2B**). We defined the merged dataset as training set, and **Table 1** displayed the detailed features of the 568 patients in the training set. Based on the expression levels of each CRGs, we performed survival analysis in the training set (**Supplementary Figures S1B–K**). The findings revealed that the expression levels of 10 CRGs were related to survival outcomes of patients, with higher expression of CDKN2A, FDX1, LIPT1 and PDHA1 being associated with a better prognosis, and higher levels of the remaining six genes suggesting a poor prognosis.

**Table 1:** The detailed characteristic of the 568 patients in the training set.

	Overall	GSE13507	TCGA	*p*
	568	165	403	
Status = Alive/Dead (%)	321/247(56.5/43.5)	96/69 (58.2/41.8)	225/178(55.8/44.2)	0.642
Age (mean (SD))	67.19(11.06)	65.18(11.97)	68.01(10.57)	0.0087
Gender = Female/Male (%)	136/432(23.9/76.1)	30/135 (18.2/81.8)	106/297(26.3/73.7)	0.04
Grade (%)				<0.001
High Grade	440(77.5)	60 (36.4)	380(94.3)	
Low Grade	125 (22)	105 (63.6)	20(5)	
Unknown	3(0.5)	0 (0.0)	3(0.7)	
T (%)				<0.001
T1	83(14.6)	80 (48.5)	3(0.7)	
T2	149(26.2)	31 (18.8)	118(29.3)	
T3	212(37.3)	19 (11.5)	193(47.9)	
T4	66(11.6)	11 (6.7)	55(13.6)	
Ta	24(4.2)	24 (14.5)	0(0.0)	
Unknown	34(6)	0 (0.0)	34(8.4)	
N (%)				<0.001
N0	383(67.4)	151 (91.5)	234(58.1)	
N1	54(9.5)	8 (4.8)	46(11.4)	
N2	80(14.1)	4 (2.4)	74(18.3)	
N3	8(1.4)	1 (0.6)	7(1.7)	
N_X_	37(6.5)	1 (0.6)	36(8.9)	
Unknown	6(1.1)	0 (0.0)	6(1.5)	
M (%)				<0.001
M0	352(62)	158 (95.8)	194(48.1)	
M1	18(3.2)	7 (4.2)	11(2.7)	
M_X_	195(34.3)	0 (0.0)	195(48.4)	
Unknown	3(0.5)	0 (0.0)	3(0.7)	
CSS Score (median [IQR])	(-0.41[-2.33, 1.53])	(-0.13[-1.58, 1.52])	(-0.53[-2.42, 1.63])	0.29
Group = High/Low (%)	284/284(50/50)	89/76(53.9/46.1)	195/208(48.4/51.6)	0.267

Next, we performed unsupervised consensus clustering algorithm. According to **Figure 2C**, k=4 was the optimum option for clustering the training set. The rationality of the grouping was verified by CDF curves and scree plot, and the details of grouping were displayed by track plot (**Supplementary Figures S2A–L**). We identified four unique patterns of CRGs and named them cuproptosis clusters1-4 respectively, including 149 cases in cluster 1, 157cases in cluster 2, 131 cases in cluster 3 and 131 cases in cluster 4. Furthermore, we discovered the obvious variances in mRNA transcriptional levels of CRGs between clusters using PCA (**Figure 2D**). As shown in **Figure 2E**, the expression of CDKN2A was higher in cluster 1 and 3, as well as the expression of LIAS, LIPT1, FDX1, MTF1, DLD and DLAT were down-regulated in cluster 1. The levels of LIAS, LIPT1 and GLS were decreased in cluster 3 compared to other clusters, while cluster 4 had the reverse pattern. In addition, the TNM categories were higher in cluster 2, 3 and 4, but there was no apparent difference in gender and age among the clusters. According to survival analysis, patients in cluster 1 had a better overall survival (OS) than the remaining groups (**Figure 2F**).

### 3.3 Cell infiltration characteristics in TME of distinct cuproptosis clusters

**Figure 3:**
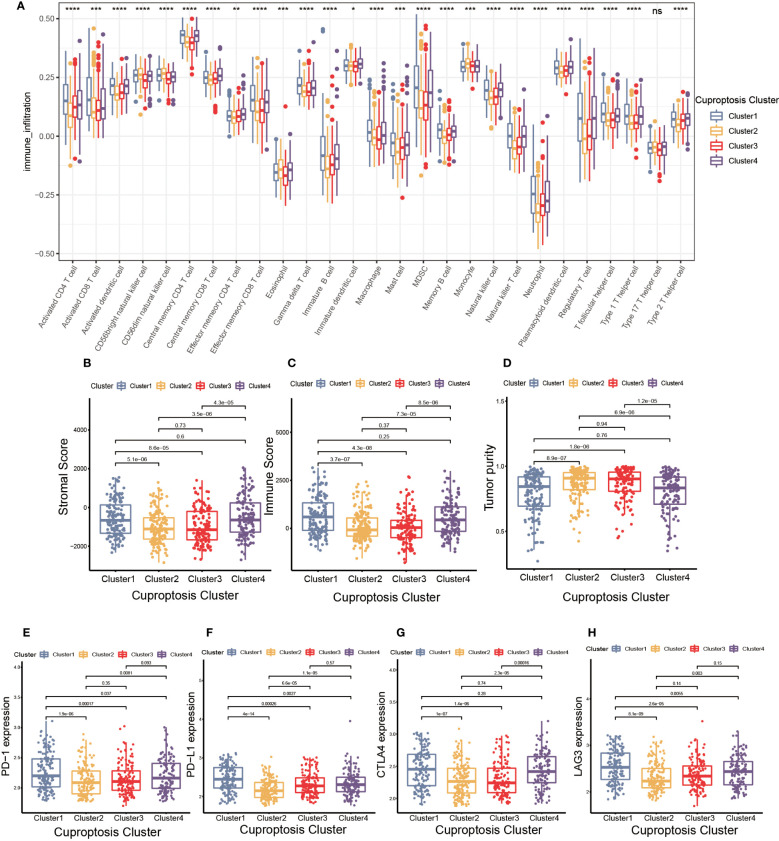
The cell infiltration characteristics in tumor microenvironment (TME) of four cuproptosis clusters. **(A)** The comparison of the abundance of immune cells infiltration in four cuproptosis clusters by single sample gene set enrichment analysis (ssGSEA) (*p< 0.05, **p< 0.01, ***p< 0.001, ****p< 0.0001; ns, no significance). **(B–D)** The stromal score **(B)**, immune score **(C)**, and tumor purity **(D)** of four cuproptosis clusters. **(E–H)** The differences in the expression levels of PD-1 **(E)**, PD-L1 **(F)**, CTLA-4 **(G)** and LAG-3 **(H)** among four cuproptosis clusters.

TME was composed of non-malignant cells, immune cells, blood vessels, nerves and lymphoid tissues which located in the center or edge of tumor lesions (33). Of these, the interaction between immune cells and tumor cells was essential for tumor development (34). Therefore, we first examined the immune cell infiltration characteristics in each cluster using the ssGSEA algorithm. As shown in **Figure 3A**, nearly all immune cells infiltrated differently in various clusters. The majority of tumor-infiltrating lymphocytes (TILs), including activated CD4^+^T cells, activated CD8^+^T cells, activated dendritic cells (DC), γδT cells, macrophages and natural killer (NK) cells were significantly enriched in cuproptosis cluster 1 and 4 compared to other clusters. Additionally, myeloid derived suppressor cell (MDSC) and regulatory T cells (Tregs) displayed the same infiltration characteristics. We also performed CIBERSORT and XCELL algorithm to predict the relative abundance of infiltrated immune cells, and found similar results (**Supplementary Figures S3A, B**). The results of CIBERSORT indicated the M1 was primarily macrophages infiltrating in TME of cuproptosis clusters 1 and 4. The findings of XCELL algorithm revealed the differences among clusters in the matrix components, such as keratinocytes, endothelial cells and neurons. The stromal score and immune score may be indicators of stromal cell and immune cell infiltrated characteristics in TME (35). As shown in **Figures 3B, C**, cuproptosis cluster 1 and 4 exhibited higher stromal score and immune score compared to the remaining clusters. Tumor purity referred to the proportion of tumor cells in tumor lesions, which reflected the features of TME (36). Cuproptosis cluster 1 and 4 obtained the lower tumor purity as a result of extensive immune and stromal cells infiltration (**Figure 3D**). In clinical immunotherapy for malignancies, the molecules PD-1, PD-L1, cytotoxic T-lymphocyte-associated protein 4 (CTLA-4) and Lymphocyte-activation gene 3(LAG-3) were extensively applied. According to **Figures 3E–H**, cuproptosis cluster 1 had the highest expression abundance of immune checkpoint among all clusters. Based on these findings, we observed the four clusters of cuproptosis pattern showed various cell infiltration characteristics in TME, which suggested different prognosis and immunotherapy effects.

### 3.4 Generation and functional annotation of DEGs based on cuproptosis cluster

**Figure 4:**
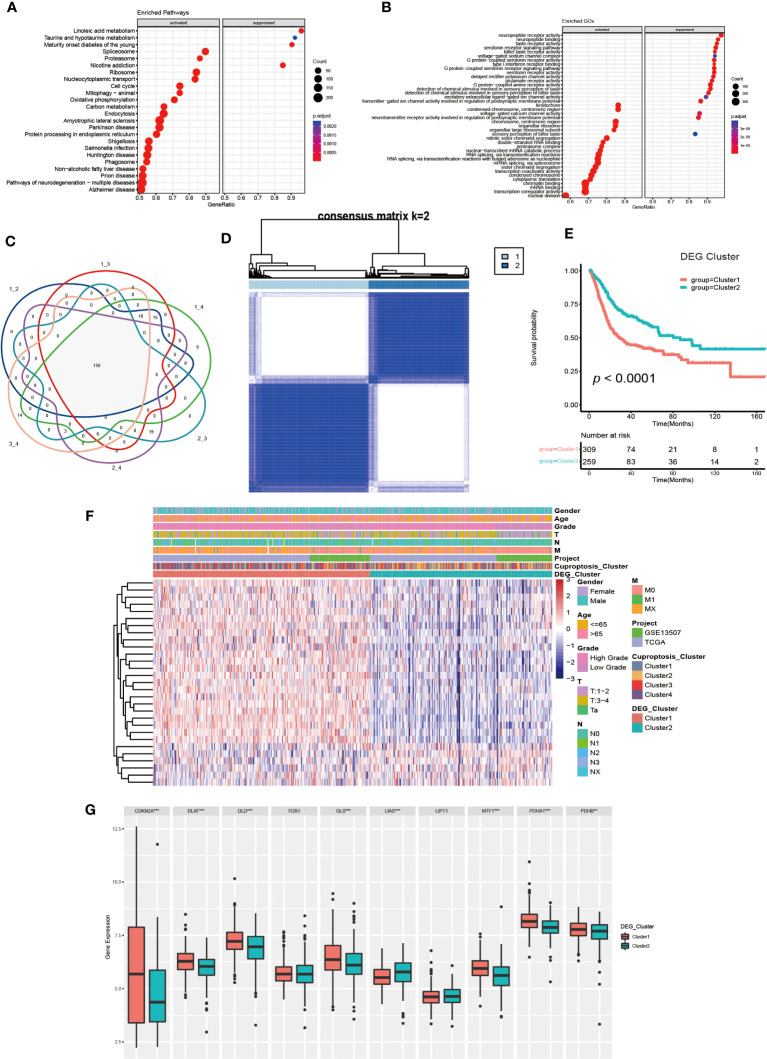
Generation and functional annotation of differentially expressed genes (DEGs) based on cuproptosis cluster. **(A, B)** The functional annotation for cuproptosis cluster-related DEGs using KEGG **(A)** and GO **(B)** enrichment analysis. **(C)** Cuproptosis clusters-related DEGs (N=150) shown in the Venn diagram. **(D)** The unsupervised consensus clustering of cuproptosis clusters-related DEGs (N=150) in the training set and consensus matrices for k =2. **(E)** The differences in OS between two DEG clusters by Kaplan-Meier curves. **(F)** The unsupervised consensus clustering analysis to classify patients into two different DEG subtypes and clinical characteristics between the two DEG clusters. **(G)** The expression of 10 CRGs between two DEG clusters (**p< 0.01, ***p< 0.001).

To further investigate the molecular mechanisms underlying the varied cuproptosis patterns, we obtained the DEGs by comparing every two clusters (**Figure 4C**), and performed KEGG and GO enrichment analysis on 150 DEGs. The results of KEGG enrichment indicated the DEGs had an obvious relationship with protein intracellular processing, spliceosome, proteasome, cell cycle and oxidative phosphorylation. The GO enrichment exhibited pathways involved in the replication and modification of genetic information. These findings demonstrated that cuproptosis was essential for intracellular signaling in TME (**Figures 4A, B**).

Next, we utilized univariate COX regression analysis on the 150 DEGs, and ultimately screened out 29 DEGs with prognostic value. The unsupervised consensus clustering algorithm was performed again on selected DEGs (N=29, **Supplementary Table S2**), and K=2 was determined to be the optimal grouping by comprehensive judgment. The patients of the training set were further divided into two different genomic subgroups (**Figure 4D**). Through calculating CDF curves and screen plot, the rationality of grouping was confirmed. Analyzing track plot visualized the details of clustering (**Supplementary Figures S4A–L**). We defined the two distinct subgroups as DEG cluster 1 and 2, with 309 patients were assigned to cluster 1 and 259 patients to cluster 2. According to survival analysis, we discovered the patients in DEG cluster 2 had a better prognosis (**Figure 4E**). According to **Figure 4F**, the expression levels of most DEGs were up-regulated in cluster1, and the patients had higher TNM categories and pathological grades. The majority of patients in DEG cluster1 were from cuproptosis cluster 3 and 4, which was consistent with previous results. Furthermore, we found the expression of several CRGs were elevated in DEG cluster1, especially the cuproptosis-facilitating genes (**Figure 4G**).

Finally, we performed a Gene Set Variation Analysis (GSVA) analysis between two different clusters. The results showed that DEG cluster1 significantly enriched pathways related to metabolism, tumor and mismatch repair, such as citrate cycle, TCA cycle, amino sugar and nucleotide sugar metabolism, glioma, renal cell carcinoma, colorectal cancer, etc. DEG cluster 2 enrichment exhibited in pathways related to drug metabolism and lipid biosynthesis. Analysis of GO enrichment showed that DEG cluster 1 had a tightly relationship with in metabolic process, protein localization and cell cycle regulation, which demonstrated that those cuproptosis-related DEGs might influence UCB progression by regulating metabolic and cell cycle-related molecular processes and pathways (**Supplementary Figures S5A–D**).

### 3.5 Construction and validation of cuproptosis scoring system

**Figure 5:**
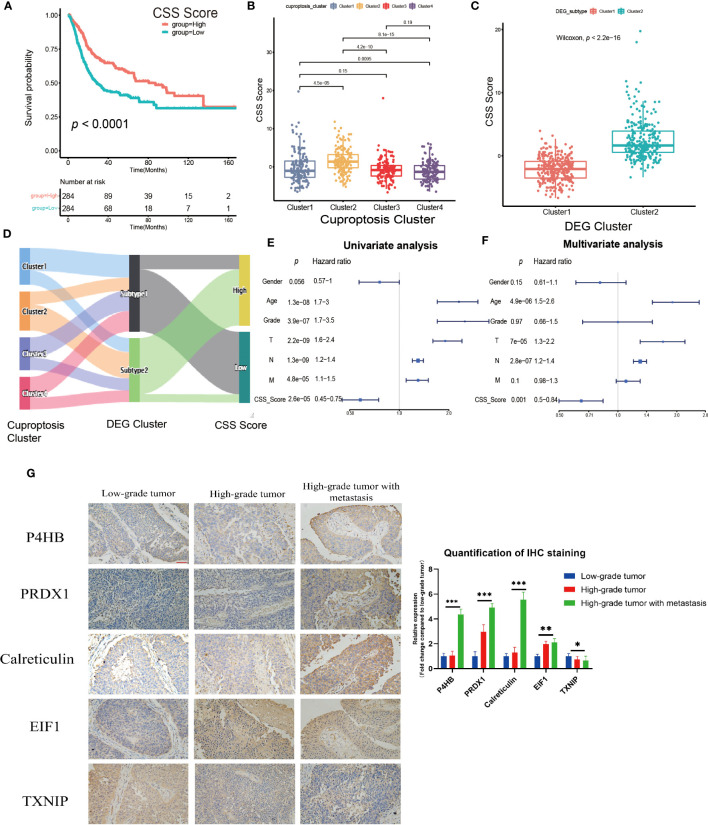
Construction and validation of the cuproptosis scoring system (CSS) model in the training set. **(A)** Survival analysis between high and low CSS score groups by Kaplan–Meier curves. **(B)** The differences in the CSS score among four cuproptosis clusters. **(C)** The differences in the CSS score between two DEG clusters. **(D)** Sankey diagram visualizing the changes of cuproptosis cluster, DEG cluster and CSS score. **(E, F)** The univariate **(E)** and multivariate **(F)** Cox regression analysis of OS. **(G) **Validation the expression of the prognostic DEGs in tumors with various pathological grades by immunohistochemical staining (magnification 200×, the scale bar is 100μm, *p< 0.05, **p< 0.01, ***p< 0.001).

The previous studies clearly linked cuproptosis to the immune and metabolic environment of tumors in the UCB patient population. However, considering the complexity among individuals and the heterogeneity of cuproptosis in various cell infiltration characteristics in TME, after comprehensively analyzed the expression of selected DEGs (N=29), we constructed the CSS model to evaluate cuproptosis patterns in individual tumor patients. Combining CSS model risk maps and Kaplan-Meier curves, we observed the patients with higher scores had a better survival (**Figure 5A**, **Supplementary Figure S5E**). The cuproptosis cluster 2 and the DEG cluster 2 exhibited higher CSS scores compared to other clusters respectively, which was in accordance with prior findings (**Figures 5B, C**). The Sankey diagram visualized the attribute changes of individual patients while linking the cuproptosis cluster, DEG cluster and CSS score (**Figure 5D**). According to the results of univariate and multivariate regression analysis, the CSS model might be as an independent prognostic factor for UCB patients (**Figures 5E, F**). Besides, we further verified the stability and efficacy of CSS model by experiments. Based on the results of univariate COX regression analysis (**Supplementary Table S2**) for selecting DEGs related to prognosis, the expression levels of genes with hazard ratio (HR) value greater than 1, such as P4HB, PRDX1, Calreticulin and EIF1, were associated with poor survival outcomes in patients of UCB. Genes like TXNIP had HR values under 1 indicating the exact opposite. The results of IHC staining was consistent with this analysis, further supporting that the CSS model had reliable efficacy in predicting prognosis of UCB patients (**Figure 5G**). To better understand the biological functions of DEGs *in vitro*, we performed cell proliferation and migration assays. After designed and synthesized the sh-RNA sequences which specifically silenced target genes (P4HB, PRDX1and Calreticulin), we validated the knockdown efficiency by qRT-PCR and selected the best one for subsequent experiments (**Supplementary Figures S6A, B**). By CCK-8 assay and colony formation assay, we discovered the proliferative capacity of bladder cancer cells significantly reduced when the expression levels of target genes were downregulated. The number of migration cells in knockdown group were dramatically lower than control group, according to transwell migration assay (**Supplementary Figures S6C–H**). The findings revealed the function of target genes in promoting tumor growth and metastasis. Additionally, we created ROC curves and calculated the AUC at different time points (**Supplementary Figure S5F**), as well as used the GSE19915 dataset as an independent validation set (**Supplementary Figures S7A–D**). These results demonstrated the CSS model had a significant prediction potential.

**Figure 6:**
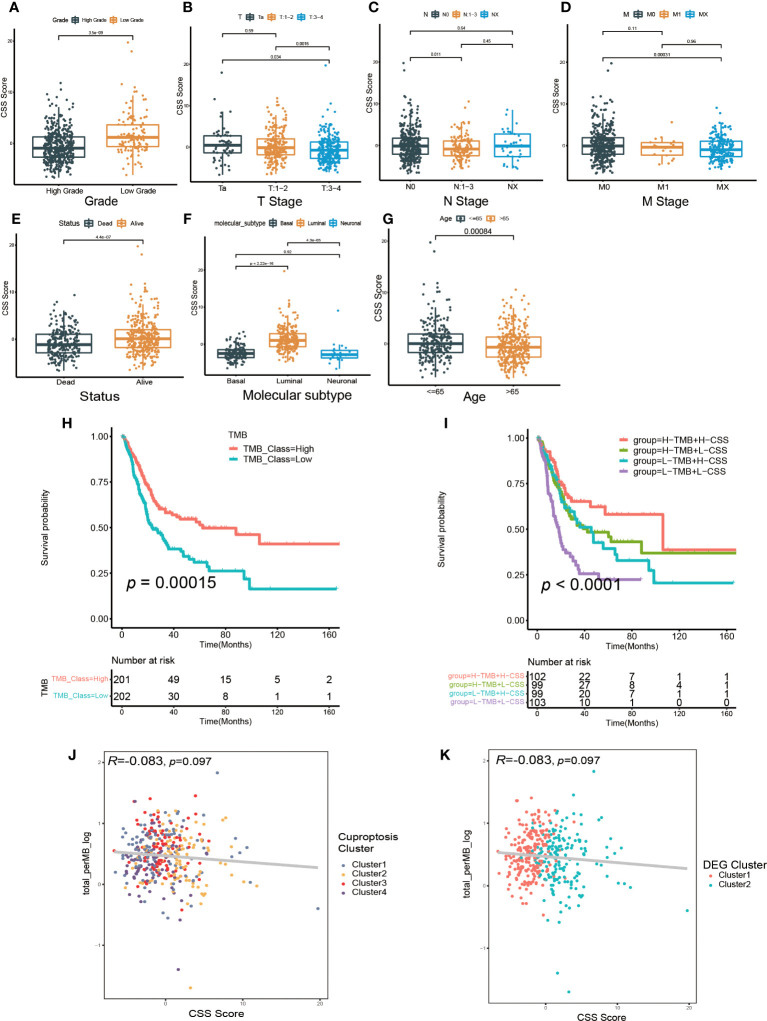
The relationship between CSS model and clinicopathological features, as well as tumor somatic mutation. **(A–G)** The differences in high and low CSS score groups among various clinical feature subgroups of grade **(A)**, T stage **(B)**, N stage **(C)**, M stage **(D)**, survival status **(E)**, molecular type **(F)**, age **(G)**. **(H) **Survival analysis for low and high TMB patient groups in TCGA-BLCA cohort using Kaplan–Meier curves. **(I)** Survival analysis for four groups classified according to TMB and CSS score in TCGA-BLCA cohort using Kaplan–Meier curves. **(J)** Linear regression analysis for tumor mutational burden (TMB) and CSS score. The dot represented each sample, and the color of the dot represented cuproptosis cluster. **(K)** Linear regression analysis for TMB and CSS score. The dot represented each sample, and the color of the dot represented DEG cluster.

Then, we investigated the relationship between CSS model and clinical characteristics of UCB patients. After statistical analysis, we found the CSS scores were linked with the grade, TNM category, final survival status, molecular type and age (**Figures 6A–G**, **Supplementary Figures S8A–G**). Specifically, patients with higher CSS scores were younger, had lower tumor grade, lower TNM categories, and longer survival times. Moreover, the luminal subgroup had the highest CSS score, followed by neuronal subgroup and basal subgroup. However, there was no significant difference of CSS score with age (**Supplementary Figures S8H, I**). Furthermore, we performed the survival analysis based on CSS scores and clinical features, and the results indicated the CSS model was a reliable survival predictor for patients with all ages and T stage classifications, especially for male patients with high grade and low NM category (N0, M0) (**Supplementary Figures S9A–O**).

### 3.6 CSS model was associated with tumor mutation burden and cell infiltration characteristics in TME

We further investigated the relationship between the CSS model and molecular mutation as well as cell infiltration features in TME. First, we discovered the patients with elevated TMB had a better prognosis (**Figure 6H**). Although the regression analysis revealed there was no significant correlation between the CSS score and TMB (**Figures 6J, K**), the combination of TMB and CSS model exhibited powerful predictive efficacy for clinical outcomes in UCB patients (**Figure 6I**).

**Figure 7:**
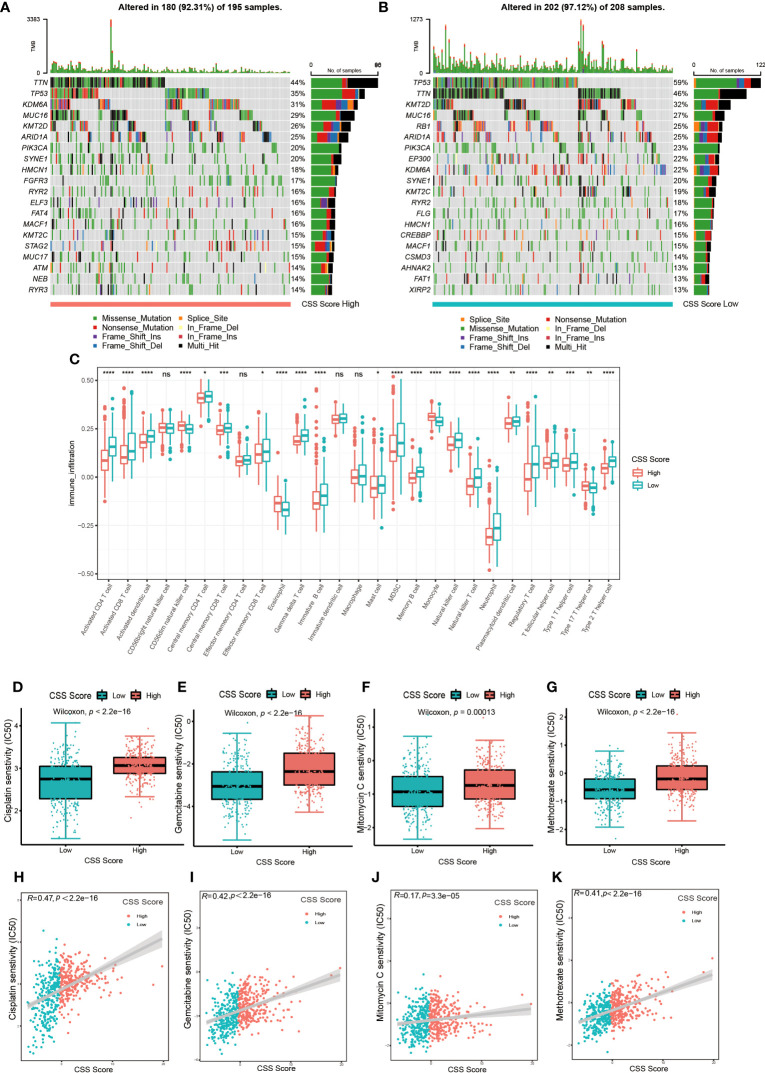
The connection between CSS model and cell infiltration characteristics in TME, as well as chemotherapy response. **(A, B)** The waterfall plot of tumor somatic mutation of high CSS score group **(A)** and low CSS score group **(B)**. **(C)** The comparison of the abundance of immune cells infiltration in high and low CSS score groups by ssGSEA (*p< 0.05, **p< 0.01, ***p< 0.001, ****p< 0.0001; ns, no significance). **(D–G)** The differences in half-maximal inhibitory concentration (IC50) of chemotherapy drugs between high and low CSS score groups in the training set. Cisplatin **(D)**, Gemcitabine **(E)**, Mitomycin C **(F)**, Methotrexate **(G)**. **(H–K)** Linear regression analysis for chemotherapy drugs sensitivity and CSS score. Cisplatin **(H)**, Gemcitabine **(I)**, Mitomycin C **(J)**, Methotrexate **(K)**.

Next, we examined how somatic mutation distribution varied between the high and low CSS score subgroups. There was no significant difference in the TMB distribution between the groups with high and low CSS score, according to comparison of **Figures 7A, B**. However, there was still a more than 10% variation in the mutation frequencies of KDM6A, TP53 and RB1, three of the most frequently mutated genes in MIBC (37). While TP53 and RB1 had higher mutation frequencies in low CSS score group, KDM6A had a higher mutation frequency in high CSS score group. **Supplementary Figure S7E** exhibited the distribution of genes with CNV alterations on chromosomes. We discovered the frequency and amplitude of gain and loss were similar between the two groups.

The results of ssGSEA revealed that the characteristics of immune cell infiltration in TME varied across high and low CSS score groups. Almost all immune cell types, including TILs, neutrophils, MDSC and Tregs, were infiltrated more in patients with lower CSS scores (**Figure 7C**). The analysis by executing CIBERSORT and XCELL algorithms suggested similar results (**Supplementary Figures S10A, B**). We also performed KEGG enrichment analysis between high and low CSS score groups. As shown in **Supplementary Figure S7F**, the group with high CSS score considerably enriched pathways associated with drug metabolism Cytochrome P450, lipid biosynthesis and metabolism pathways. The group with low CSS score enrichment exhibited in pathways related to tumor, nucleotide mismatch repair, metabolism of glucose, lipid and amino acid. These results further suggested the cuproptosis might be closely connected to immune and metabolic microenvironments in UCB patients.

### 3.7 Predictive significance of CSS model for chemotherapy and immunotherapy

First, we explored whether the CSS score was related to the chemotherapy response. We selected several frequently applied chemotherapeutic drugs in UCB, including gemcitabine, mitomycin C, cisplatin and methotrexate, then investigated the correlation between IC50 and CSS score. As shown in **Figures 7D–K**, the IC50 of above four drugs was lower in the group with low CSS score, and there was a significant positive association between the two variables. The findings demonstrated that chemotherapy might provide more therapeutic benefits for patients in low CSS score group.

**Figure 8:**
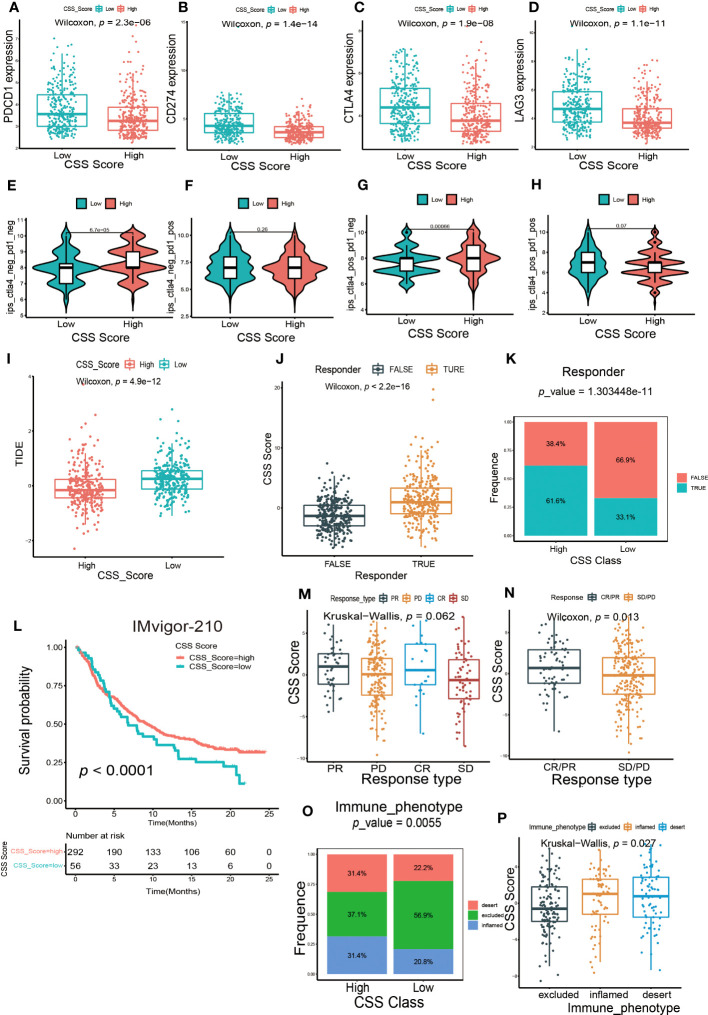
The predictive efficacy of CSS model in immunotherapy. **(A–D)** The differences in the expression of immune checkpoint between high and low CSS score groups in the training set. **(E–H)** The differences of response index between high and low CSS score groups among four subgroups. **(I)** The differences in the Tumor Immune Dysfunction and Exclusion (TIDE) score between high and low CSS score groups in the training set. **(J)** The differences in CSS scores between groups with response and non-response to immunotherapy in the training set. **(K)** The proportion of patients who response to immunotherapy in high or low CSS score groups. **(L)** Survival analysis between high and low CSS score groups by Kaplan–Meier curves in the IMVigor-210 cohort. **(M)** The differences in CSS scores among four different immunotherapy responses in the IMvigor 210 cohort. **(N)** The differences in CSS scores among two combination immunotherapy responses in the IMvigor 210 cohort. **(O)** The proportion of three immune phenotypes in high or low CSS score groups. **(P)** The differences in CSS score among immune-excluded phenotype, immune-inflamed phenotype and immune-desert phenotype.

Next, we investigated whether the CSS model could predict the outcome of immunotherapy. We found the expression of several common immune checkpoints, such as PD-1, PD-L1, CTLA-4 and LAG-3, were obviously decreased in patients with high CSS score group (**Figures 8A–D**). Depending on the usage of anti-PD-1 and anti-CTLA-4 immunotherapy, the UCB patients were divided into four distinct subgroups. As shown in **Figures 8E–H**, in patients with the CTLA4-negative PD-1 negative and CTLA-4-positive PD-1 negative subgroups, the higher CSS score was significantly associated with better immunotherapy response. However, there was no correlation between CSS score and immunotherapy efficacy in patients with the CTLA-4-negative PD-1 positive and CTLA-4-positive PD-1 positive subgroups, suggesting the cuproptosis was more closely related to the patients who had negative response against PD-1 immunotherapy. TIDE score is linked to tumor immune escape characteristics and can predict the effect of immune checkpoint blockade (ICB) therapy. We discovered that the UCB patients with lower CSS scores had higher TIDE scores (**Figure 8I**), suggesting these patients might be more vulnerable to immune escape and had an unsatisfactory therapy effect. Additionally, we found the individuals with no response to immunotherapy generally had lower CSS scores (**Figures 8J, K**). A multicenter, phase II clinical trial for patients with advanced or metastatic urothelial carcinoma using the PD-L1 inhibitor atezolizumab reported the effective outcome (IMvigor 210, NCT02108652) (38). In this cohort, we discovered that the UCB patients with high CSS scores had longer life expectancies (**Figure 8L**). The response to immunotherapy was classified into complete response (CR), partial response (PR), stable disease (SD) and progressive disease (PD). As exhibited in **Figure 8M**, patients with CR and PR had higher CSS scores. When combining CR and PR, as well as SD and PD to create an integrative outcome, the difference between CSS scores and treatment outcomes would have been more obvious (**Figure 8N**). Overall, patients in the high CSS group showed a greater therapeutic advantage and clinical response to immunotherapy. Finally, we investigated the relationship between CSS score and immunophenotype. In high CSS score group, the proportion of patients with three immunophenotypes was approximately equal. More than half of the patients in low CSS score group exhibited an immune-excluded phenotype, indicating they might have a poor response to immunotherapy (**Figure 8O**). Furthermore, we also observed the patients with immune-inflamed and immune-desert phenotypes had higher CSS scores (**Figure 8P**). Taken together, these findings demonstrated that CSS model could serve as an indicator for predicting chemotherapy and immunotherapy efficacy.

## 4 Discussion

The crucial function of copper in tumorigenesis has gradually been recognized with the advancement of trace element detection technology, making it become an attractive target for the development of novel drugs. Some scholars have proposed that copper may be a vulnerable point in the progression of cancer (29). Cuproptosis, as a novel and unique mechanism of cell death, was considered to have potential value in cancer treatment. Ji et al. (39) found cuproptosis was associated with development of kidney renal clear cell carcinoma (KIRC) and established a score system based on CRGs for predicting prognosis. Zhang et al. (40) reported FDX1, the key regulator of cuproptosis, was down-regulated in hepatocellular carcinoma (HCC) and correlated with longer OS. Furthermore, Yang et al. (41) identified eight cuproptosis-related lncRNAs signature of head and neck squamous cell carcinoma (HNSC) as prognostic indicator. Previous studies concentrated on the prognostic value of CRGs, but neglected to deeply explorer the comprehensive effect of cuproptosis in tumors and its interaction with cell infiltration in TME, which was significant for the application of cuproptosis in clinical therapy strategies.

In our study, we initially discovered the differently expression of CRGs in UCB samples with various grades. We observed the expression of CDKN2A was significantly elevated in high-grade samples, and it had the highest frequency of somatic mutation and copy number loss. As a tumor suppressor gene, its mutation has been identified as a risk factor for tumorigenesis and development of MIBC (42). Additionally, CDKN2A was a crucial cuproptosis-facilitating gene, indicating the cuproptosis might related to regulate the progression of UCB. Then, we identified four different cuproptosis clusters, and found the differences among clusters in TME immune cell infiltration characteristics, clinical features and prognostic of patients. Cuproptosis cluster1 could be considered as “hot” tumor, because it infiltrated the largest number of immune cells in TME, as well as the patients had better survival status and clinicopathological characteristics. Cuproptosis cluster 2 and 3 characterized by lacking immune cells infiltration in TME, especially the patients in cluster 3 were more likely to have poorer prognosis. Surprisingly, patients in cuproptosis cluster 4 had worse prognosis despite a majority of immune cells infiltration in TME. We discovered there were abundant MDSCs and Tregs simultaneously, which are crucial immunosuppressive cells that considerably inhibited the infiltration and function of CTLs (43), and we inferred they might predominate in TME of cuproptosis cluster 4. Depending on the status of tumor immune response, TME could be divided into three immune phenotypes: immune-inflamed phenotype, immune-excluded phenotype and immune-desert phenotype (44). The immune-inflamed phenotype generally referred to tumors with a majority of activated immune cells infiltrated in TME, particularly CTLs. The immune-excluded phenotype also infiltrated a variety types of immune cells, but the strong inhibitory microenvironment prevented cells to penetrate the stroma into tumor, resulting in lack of immune cells infiltration in the tumor stroma and parenchyma, meanwhile limiting the migration and function of CTLs. Therefore, cuproptosis cluster 1 was attributed to immune-inflamed phenotype and expected to benefit more from immunotherapy. Cuproptosis cluster 2 and 3 belonged to immune-desert phenotype, and cuproptosis cluster 4 belonged to immune-excluded phenotype, both of which responded weakly to immunotherapy (45). In the immune-excluded phenotype, transforming growth factor beta (TGF-β) convert the TME into an inhibitory environment by suppressing the activity of anti-tumor immune cells, while simultaneously enhancing the inhibitory function of Tregs, which was considered to be the primary immunosuppressive factor (46, 47). In addition, cytokines such as IL-8 and IL-6 also had a significant inhibitory effect (48). The results of GSVA enrichment analysis showed that cuproptosis cluster 4 significantly enriched pathways involved in TGF-β, IL-6 and tumorigenesis, which corresponded to previous analysis (**Supplementary Figures S11A, B**). Different cuproptosis patterns had an obvious correlation with the features of immune cell infiltration in TME, indicating the immune cells and molecules were crucial in cuproptosis regulating UCB progression.

Next, we analyzed different genes between distinctive cuproptosis clusters and generated DEGs with prognostic value. After accumulating information regarding related pathways of DEGs, we inferred the cuproptosis might have impact on signaling transduction pathway. The results of cuproptosis-related DEG clusters further revealed cuproptosis was deeply involved in the progression of UCB via modulating immune cell infiltration in TME and regulating metabolic and cell cycle-related pathways. Then, we constructed the CSS model to quantify the cuproptosis patterns of individual UCB patients and validated the scoring system from multiple perspectives, which showed reliable prediction efficacy. Subsequently, we discovered the CSS model was tightly connected to several clinical characteristics, indicating the CSS model has widespread clinical application prospects.

The biological functions of DEGs related to prognosis were verified by cell proliferation and migration assays and IHC. The expression of P4HB was significantly increased in both UCB tissues and cell lines. Some researchers have suggested that P4HB could be served as a biomarker to predict the progression and prognosis of UCB (49, 50). A study had reported the UCB patients with elevated urinary PRDX1 levels were more likely to experience recurrence (51). Similarly, the quantity of Calreticulin in urine has been proposed as a diagnostic indicator for UCB (52). Since the CSS model was constructed by analyzing the expression of DEGs, it also indicated the predictive efficacy of CSS model was consistent and dependable.

TMB has been served as a potential marker for predicting response to immunotherapy and significantly associated with clinical features of patients (53, 54). Our result demonstrated the combination of TMB and CSS model was an effective biomarker for predicting prognosis of UCB patients. The mutation frequencies of some genes were slightly different between high and low CSS score groups. The deficiency of KDM6A promoted the polarization of M2 macrophages and coordinated with p53 dysfunction to cause bladder cancer (55). Additionally, Qiu et al. found that KDM6A loss triggered an epigenetic switch that disrupted urothelial differentiation and induced a neoplastic state characterized by increased cell proliferation during bladder carcinogenesis (56). Co-mutations of RB1 and TP53 were tightly related to genomic biomarkers of response to immunotherapy in UCB patients (57). Moreover, our findings revealed the connection between CSS score and immune cell infiltration features. There were a large number of immunosuppressive cells simultaneously infiltrated in TME of the patients with low CSS score. We speculated these cells had a major impact when combined with clinicopathological characteristics and prognosis data of patients. Taken together, the CSS score could serve as an indicator to evaluate the cell infiltration characteristics of individuals in TME. Combining the CSS score with biomarkers like TMB might be an efficient method for predicting the response to treatment and prognosis of patients.

Furthermore, we evaluated the potential of the CSS model to predict the sensitivity to chemotherapy of patients. The combination of gemcitabine and cisplatin is the standard first-line chemotherapy strategy in currently clinical practice, with mild adverse reaction and reliable efficacy (58). Methotrexate is the traditional chemotherapeutic drug for MIBC patients, while mitomycin C is an effective drug for intravesical chemotherapy after TURB to prevent NMIBC recurrence (59). Our result demonstrated the patients with low CSS scores would benefit more from treatment. In addition, the CSS model was a predictor for efficacy of chemotherapy, and it was reliable for selecting suitable patients and drugs for chemotherapy in order to guide clinical treatment.

ICB therapy held widespread application prospect in the treatment of various malignancies by enhancing the effectiveness of human immune cells to kill tumor cells (60). According to the recent guidelines recommended, PD-1/PD-L1 inhibitors have been approved for second-line treatment of patients with unrespectable and metastatic MIBC, and first-line treatment for platinum-intolerant and PD-L1-positive patients, which benefit from clinical treatment (61). Despite this, a significant portion of patients discontinued the immunotherapy because of serious side effects and unfavorable treatment response. The Results of a Phase II clinical trial of patients who were advanced or metastatic urothelial carcinoma using the PD-1 inhibitor pembrolizumab (KEYNOTE-052) revealed the total effective rate was only 24% (62). Therefore, it was crucial to screen out the suitable candidates for immunotherapy. Based on the connection between CSS score and immunotherapy outcome, we discovered the patients with high CSS scores had greater immunotherapy outcomes, and PD-1 inhibitor treatment would be more effective. Additionally, we found the majority of patients with low CSS scores had immune-excluded phenotype, indicating unfavorable treatment outcomes and prognosis. Collectively, the CSS model could be used as both an effective indicator to predict immunotherapy response and a reliable signal to identify patients who would benefit from immunological treatment.

Although the study provided the unique insights in predicting the clinical outcome and immunotherapy response of UCB patients, there were still some limitations. On the one hand, all conclusions come from the processing and analysis of public database data, and more complete and comprehensive clinical data need to be collected to verify the results. On the other hand, the involved CRGs in this study were screened by genome-wide CRISPR-Cas9, and the specific roles of these genes in cuproptosis still need to be confirmed by large number of basic experiments, which would assist us in investigating the comprehensive biological functions of cuproptosis in UCB.

In conclusion, our research described and illuminated the underlying regulatory mechanisms of cuproptosis in UCB. The variations in cuproptosis patterns may significantly influence the heterogeneity of tumor clinicopathological features and TME, affecting the response to chemotherapy and immunotherapy. The CSS model was not only effective in predicting the prognosis of patient, but in assessing response to chemotherapy and immunotherapy and ultimately guiding individualized precision treatment.

## Data availability statement

The original contributions presented in the study are included in the article/**Supplementary Materials**, further inquiries can be directed to the corresponding author/s.

## Ethics statement

The studies involving human participants were reviewed and approved by Institutional Review Committee of Tongji Hospital, Tongji Medical College, Huazhong University of Science and Technology. The patients/participants provided their written informed consent to participate in this study. Written informed consent was not obtained from the individual(s) for the publication of any potentially identifiable images or data included in this article.

## Author contributions

ZD and HF designed this study, analyzed the data, performed the experiments and drafted the manuscript. YH and ZL extracted the information from the databases. RY, TW and JL supervised the entire work. All authors contributed to the article and approved the submitted version.
