# Supplementary material for: A novel cuproptosis pattern and tumor immune microenvironment characterization in urothelial carcinoma of the bladder
Source: Front Immunol. 2023 Aug 17;14:1219209. doi: 10.3389/fimmu.2023.1219209 (PMC10469981; doi:10.3389/fimmu.2023.1219209)
Supplement: Supplementary file 1 [file DataSheet_1.zip › Supplementary material information.docx]

**Supplementary Figure S1.** (A) The expression of 10 cuproptosis-regulated genes (CRGs) between normal tissues and tumor tissues in the TCGA-BLCA cohort (ns, no significance, **p* < 0.05, ***p* < 0.01). (B-K) Survival analysis according to the expression of 10 CRGs by Kaplan–Meier curves in the training set.

**Supplementary Figure S2.** The process of generating four cuproptosis cluster. (A-I) The unsupervised consensus clustering based on the expression levels of 10 CRGs in the training set and consensus matrices for k=2-9. (J) The cumulative distribution function (CDF) curve for k=2-9. (K) The scree plot for k=2-9. (L) The track plot for k=2-9.

**Supplementary Figure S3.** The comparison of immune cells infiltration characteristics in four cuproptosis clusters by CIBERSORT (A) and XCELL (B) (ns, no significance, **p* < 0.05, ***p* < 0.01, ****p* < 0.001, *****p* < 0.0001).

**Supplementary Figure S4.** The process of generating two different DEG clusters. (A-I) The unsupervised consensus clustering analysis of cuproptosis cluster related DEGs in the training set and consensus matrices for k=2-9. (J) The cumulative distribution function (CDF) curve for k=2-9. (K) The scree plot for k=2-9. (L) The track plot for k=2-9.

**Supplementary Figure S5.** (A-D) Gene set variation analysis (GSVA) enrichment analysis between two DEG clusters using KEGG datasets (A) and GO enrichment (B-D). (E) Cuproptosis scoring system (CSS) model risk maps of the training set. (F) Receiver operating characteristic (ROC) curve of the CSS model.

**Supplementary Figure S6.** Knockdown of the target genes (P4HB, PRDX1 and Calreticulin) inhibited the proliferation and migration of T24 and 5637 cells. (A, B) The efficiencies of target genes knockdown were assessed by qRT-PCR in T24 and 5637 cell lines. (C, D) The proliferation capacities of T24 and 5637 cells after target genes knockdown were detected by cell counting kit-8 (CCK-8) assay. (E, F) The clonal proliferation capacities of T24 and 5637 cells after target genes knockdown were determined by colony formation assay. (G, H) The migration abilities of T24 and 5637 cells after target genes knockdown were determined by transwell assay (magnification 200×, the scale bar is 100μm, ***p* < 0.01, ****p* < 0.001).

**Supplementary Figure S7.** (A-D) Validate the CSS model in GSE19915. (A) CSS model risk maps. (B) Survival analysis between high and low CSS score groups by Kaplan–Meier curves. (C) The univariate Cox regression analysis of OS. (D) The multivariate Cox regression analysis of OS. (E) The distribution of genes on chromosomes with CNV alteration between high (upper) and low (bottom) CSS score groups, where gains are in red, and losses are in blue. (F) GSVA enrichment analysis using KEGG dataset between high and low CSS score groups.

**Supplementary Figure S8.** (A-G) The proportion of patients with different clinical feature subgroups of grade (A), T stage (B), N stage (C), M stage (D), final survival status (E), molecular type (F), age (G) in high or low CSS score. (H) Differences in CSS score between female and male subgroups. (I) The proportion of patients with different gender in high or low CSS score groups.

**Supplementary Figure S9.** Survival analysis for patients with high or low CSS score in several subgroups of gender (A-B), age (C-D), grade (E-F), T stage (G-I), N stage (J-L), M stage (M-O).

**Supplementary Figure S10.** The comparison of immune cells infiltration characteristics in high and low CSS score groups by CIBERSORT (A) and XCELL (B) (ns, no significance, **p* < 0.05, ***p* < 0.01, ****p* < 0.001, *****p* < 0.0001).

**Supplementary Figure S11.** (A) GSVA enrichment analysis showing the activation or inhibition status of biological pathways between cuproptosis cluster 2 and 4. (B) GSVA enrichment analysis showing the activation or inhibition status of biological pathways between cuproptosis cluster 3 and 4.

**Supplementary Table S1.** The sh-RNA sequences and primers sequences (5’-3’) about target genes (P4HB, PRDX1 and Calreticulin).

**Supplementary Table S2.** The DEGs related to prognosis screened out by univariate COX regression analysis and used to construct CSS model.
