# Supplementary figures and images for: A novel cuproptosis pattern and tumor immune microenvironment characterization in urothelial carcinoma of the bladder
Source: Front Immunol. 2023 Aug 17;14:1219209. doi: 10.3389/fimmu.2023.1219209 (PMC10469981; doi:10.3389/fimmu.2023.1219209)

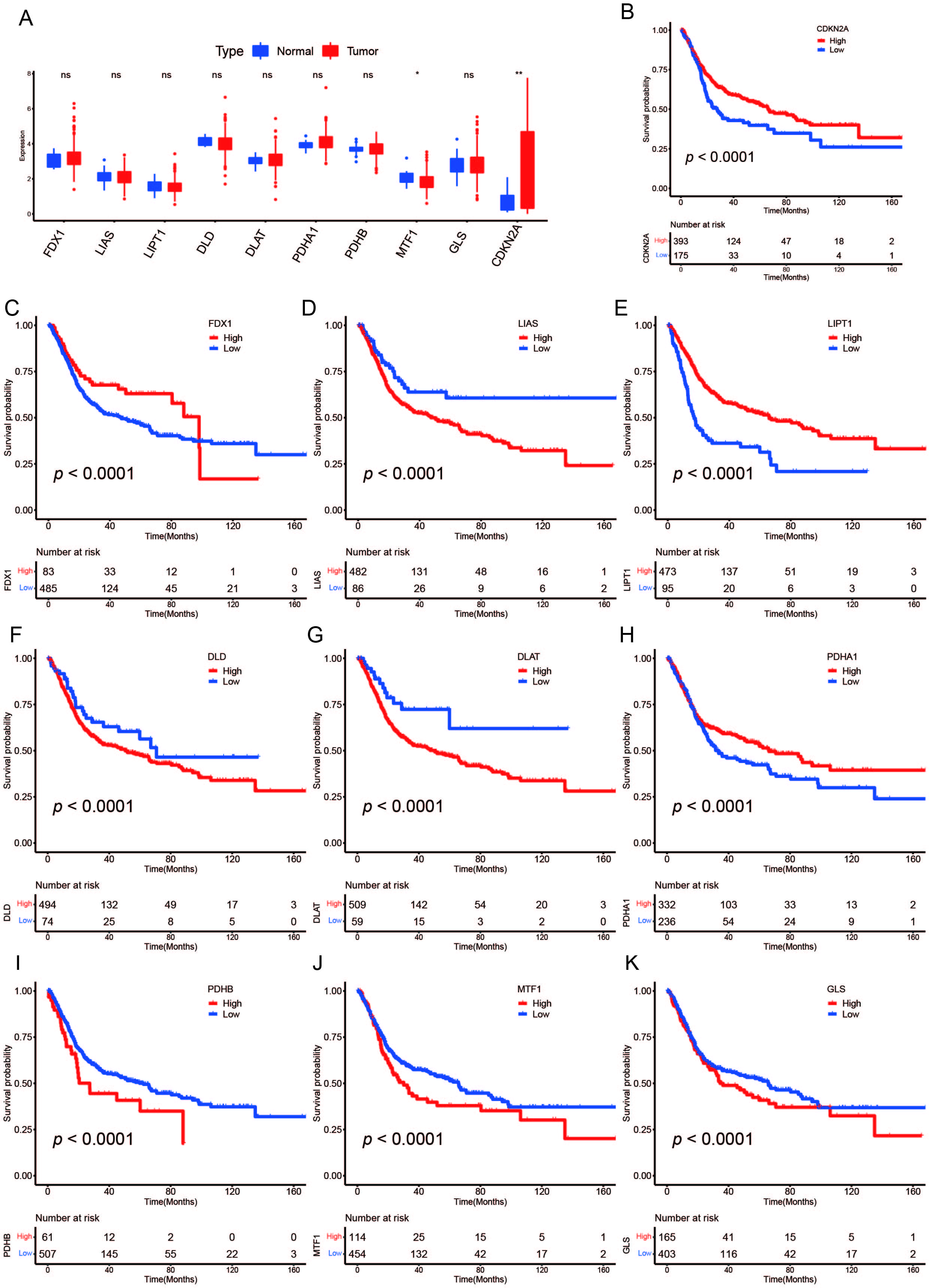

Supplement: Supplementary file 1 [file DataSheet_1.zip › Supplementary Figure S1.jpg]

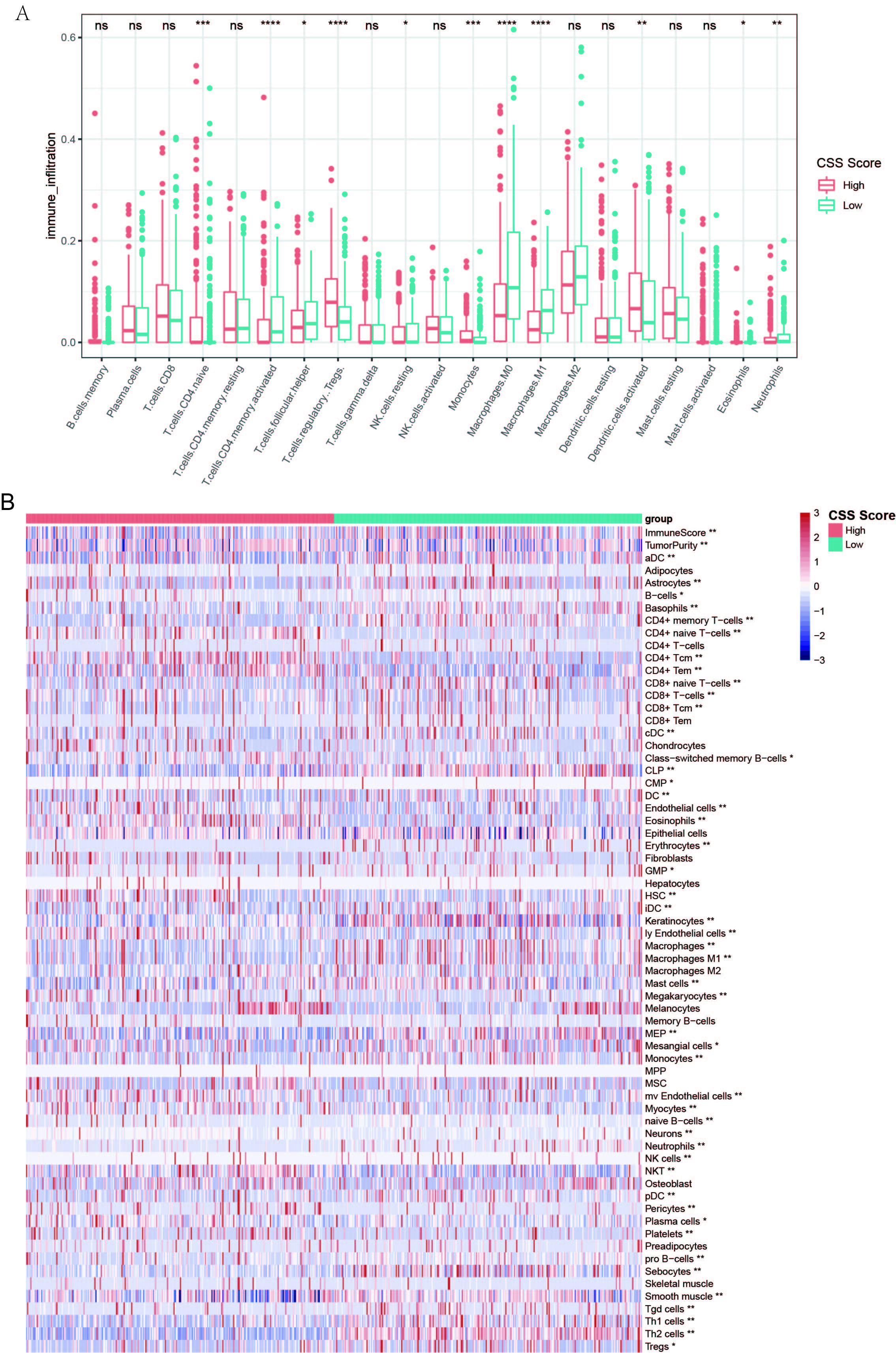

Supplement: Supplementary file 1 [file DataSheet_1.zip › Supplementary Figure S10.jpg]

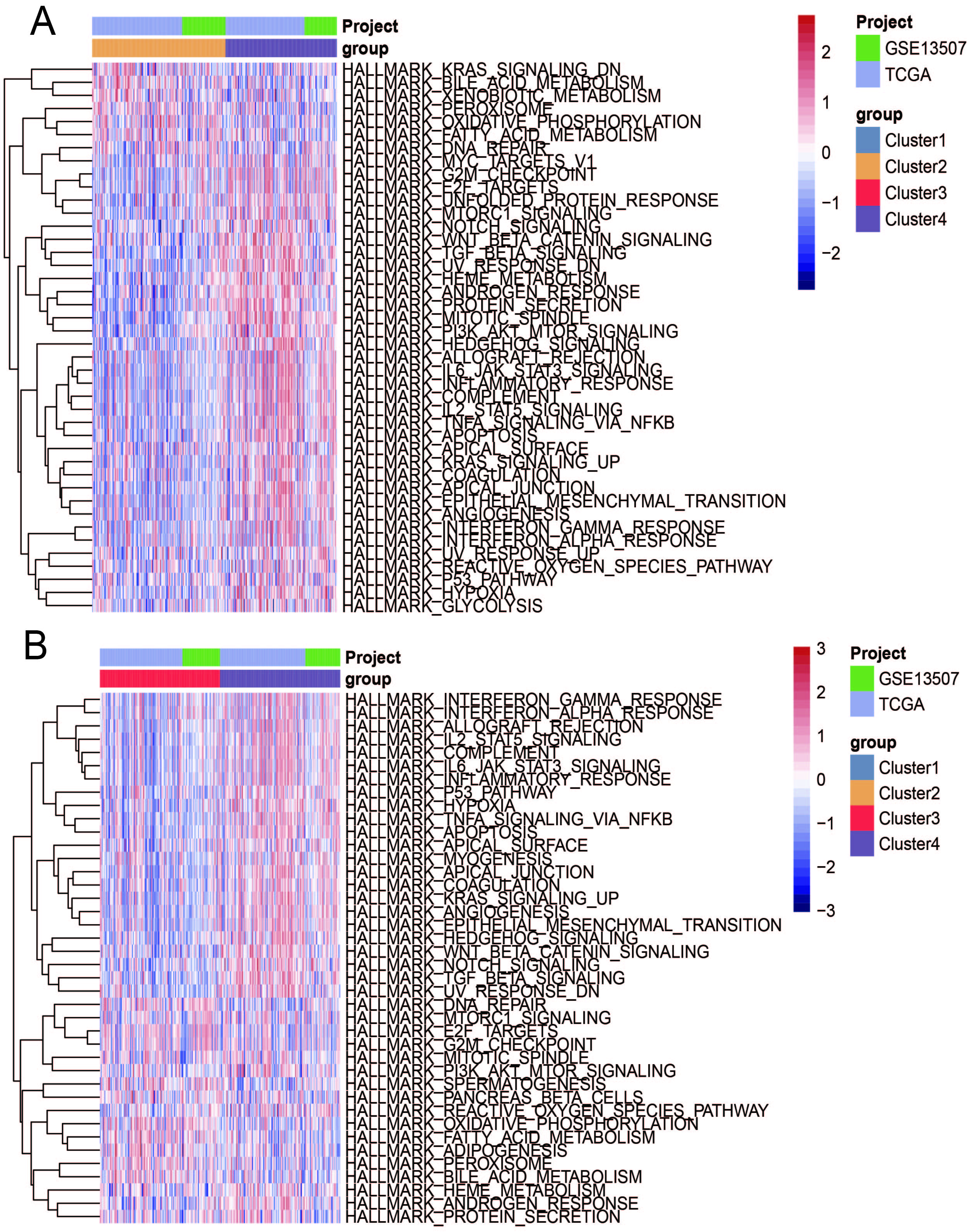

Supplement: Supplementary file 1 [file DataSheet_1.zip › Supplementary Figure S11.jpg]

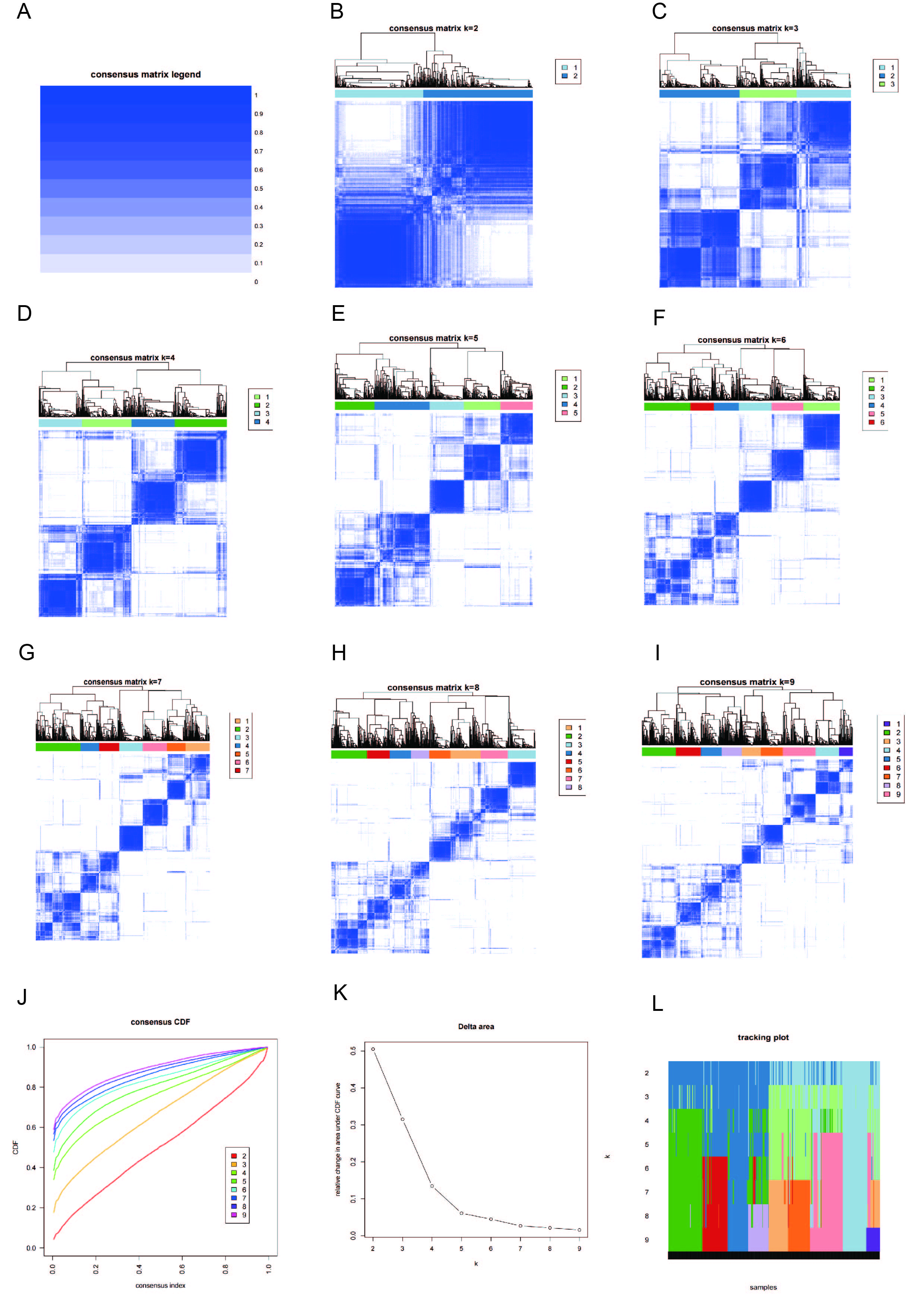

Supplement: Supplementary file 1 [file DataSheet_1.zip › Supplementary Figure S2.jpg]

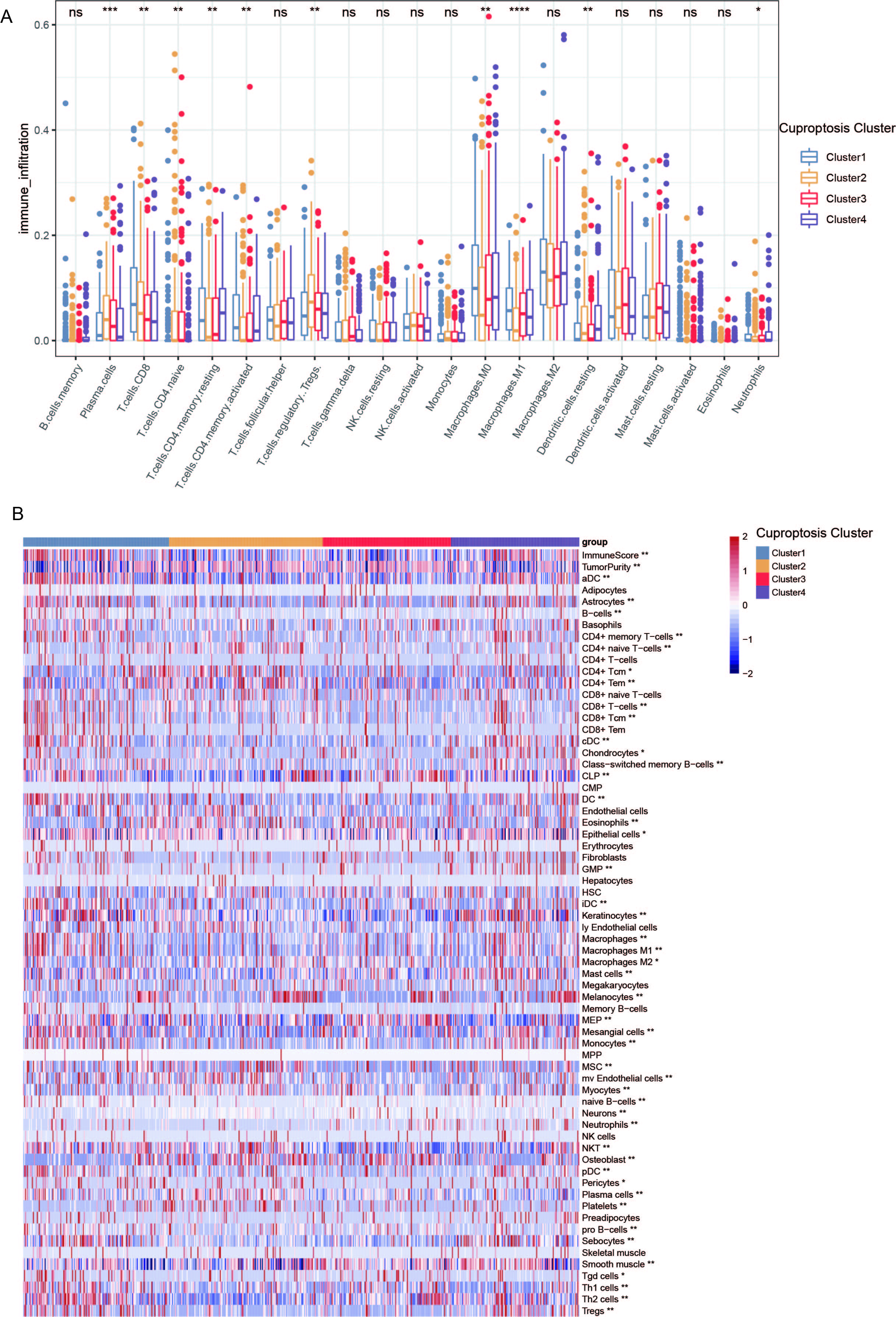

Supplement: Supplementary file 1 [file DataSheet_1.zip › Supplementary Figure S3.jpg]

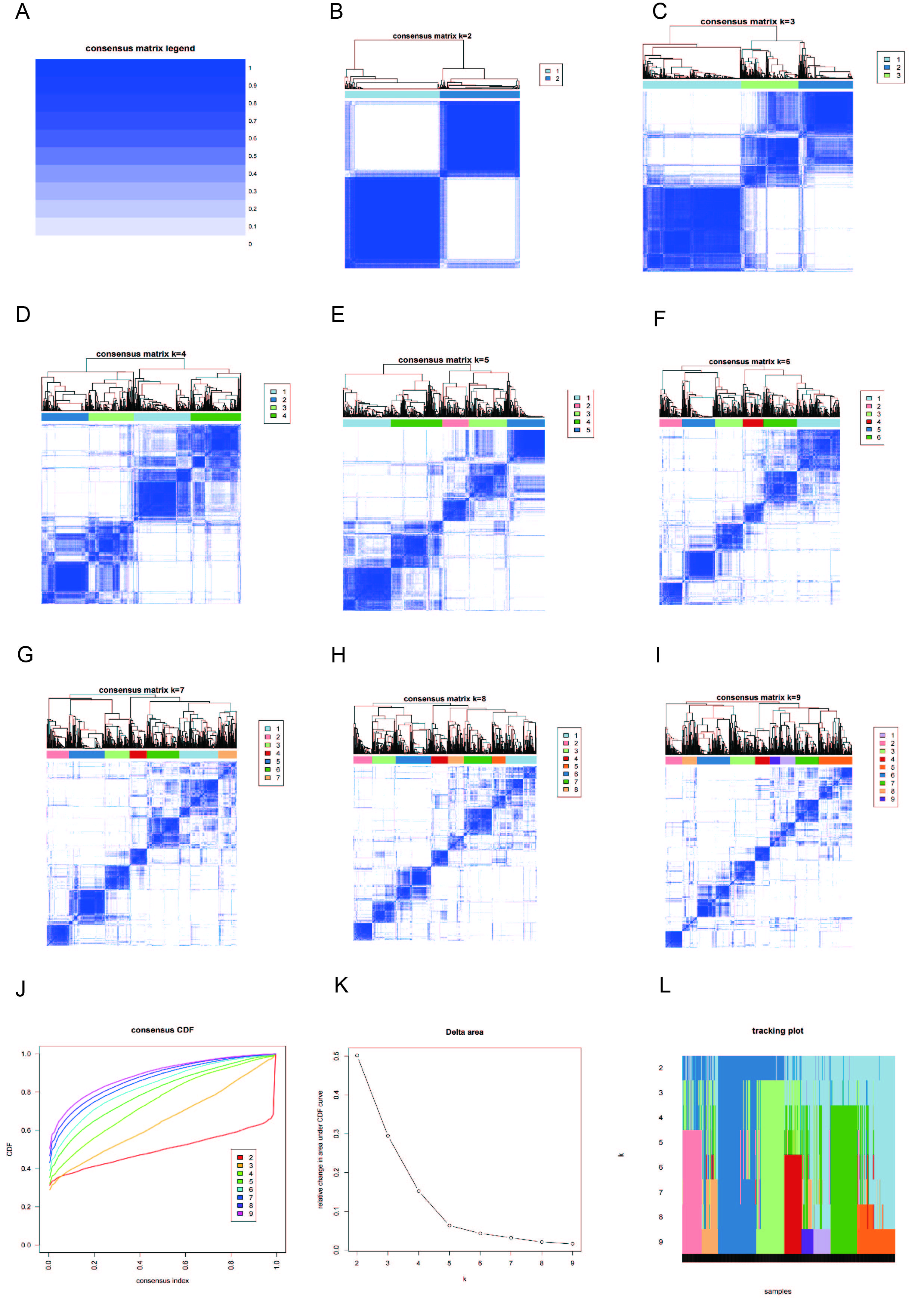

Supplement: Supplementary file 1 [file DataSheet_1.zip › Supplementary Figure S4.jpg]

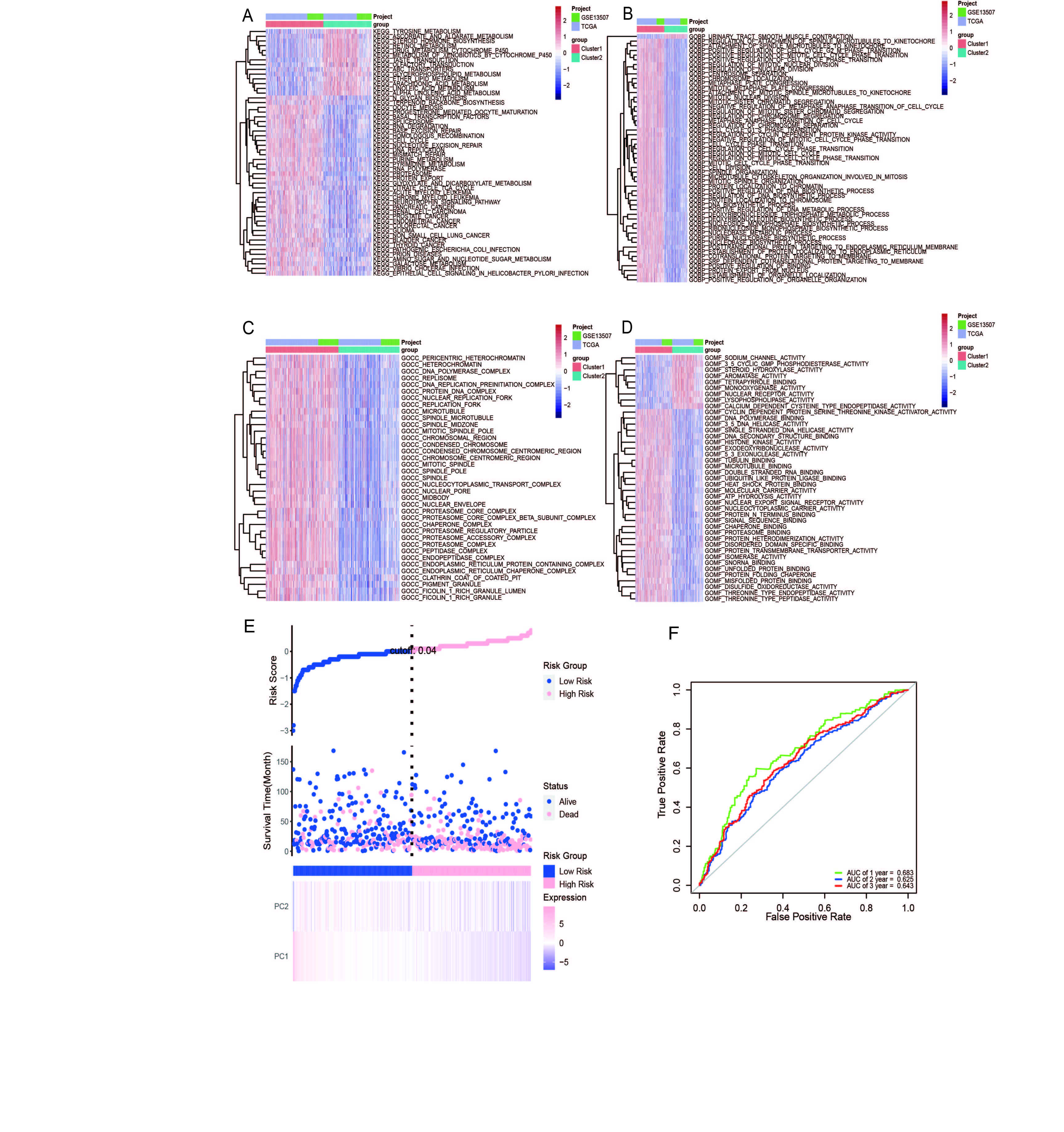

Supplement: Supplementary file 1 [file DataSheet_1.zip › Supplementary Figure S5.jpg]

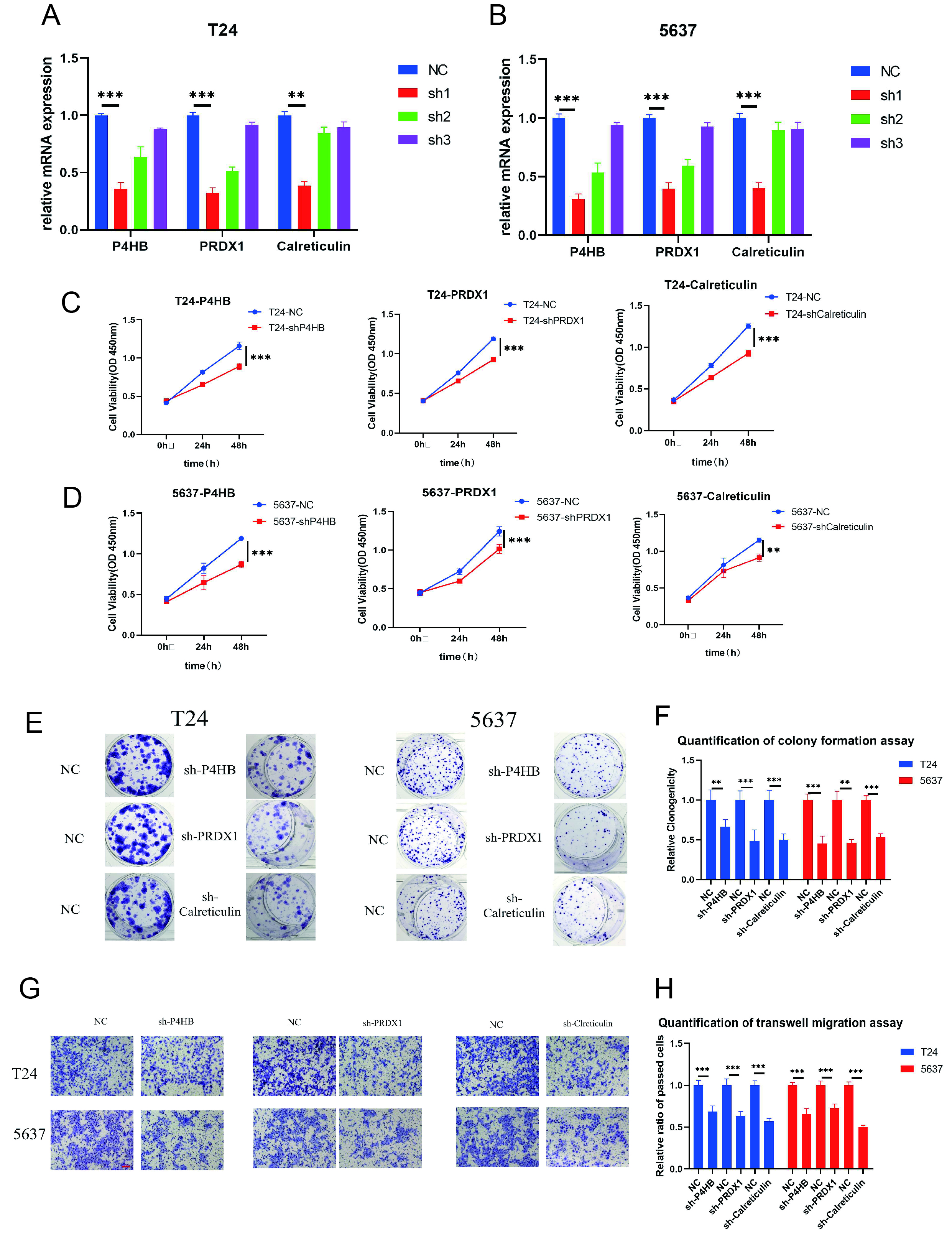

Supplement: Supplementary file 1 [file DataSheet_1.zip › Supplementary Figure S6.jpg]

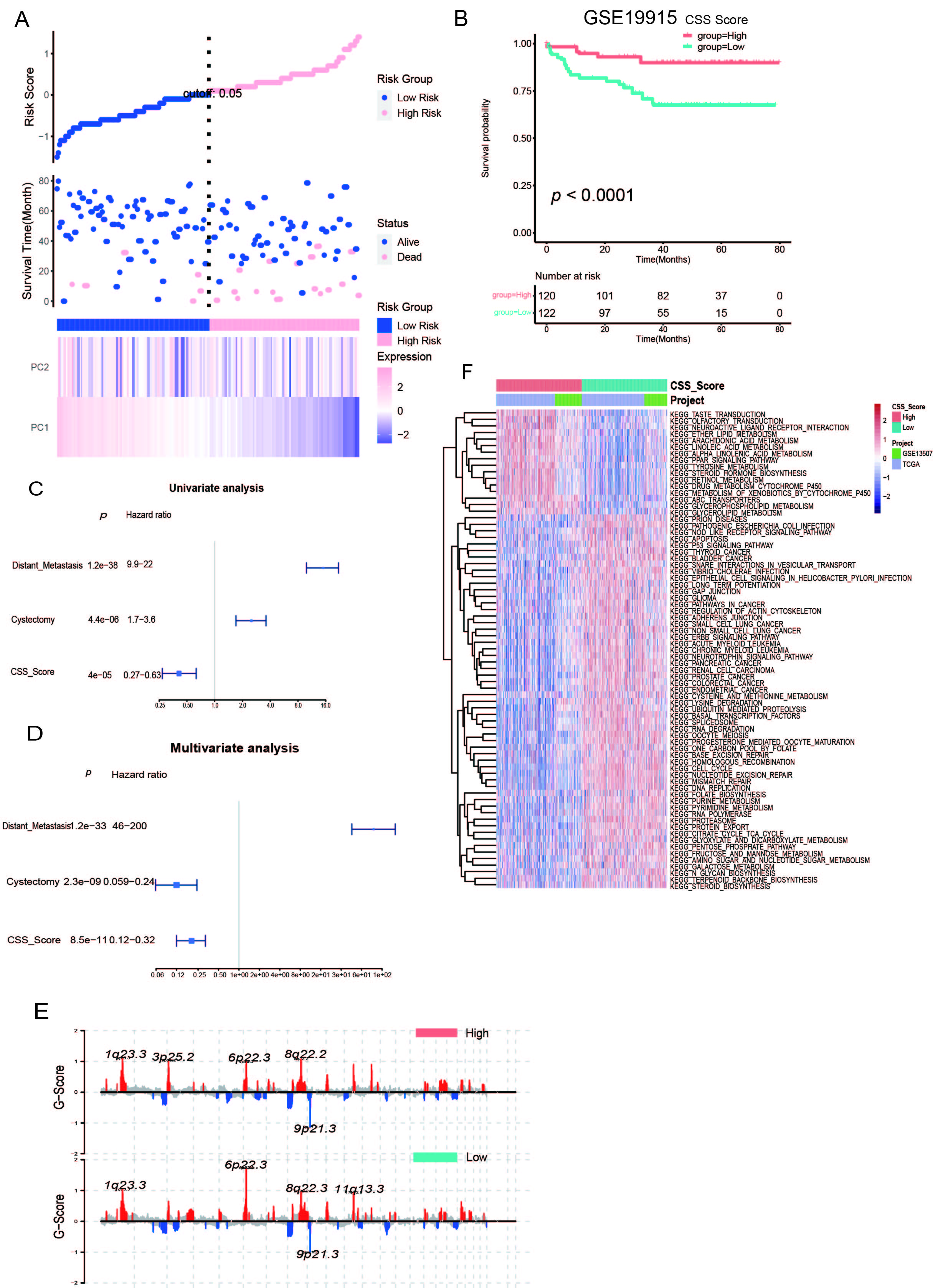

Supplement: Supplementary file 1 [file DataSheet_1.zip › Supplementary Figure S7.jpg]

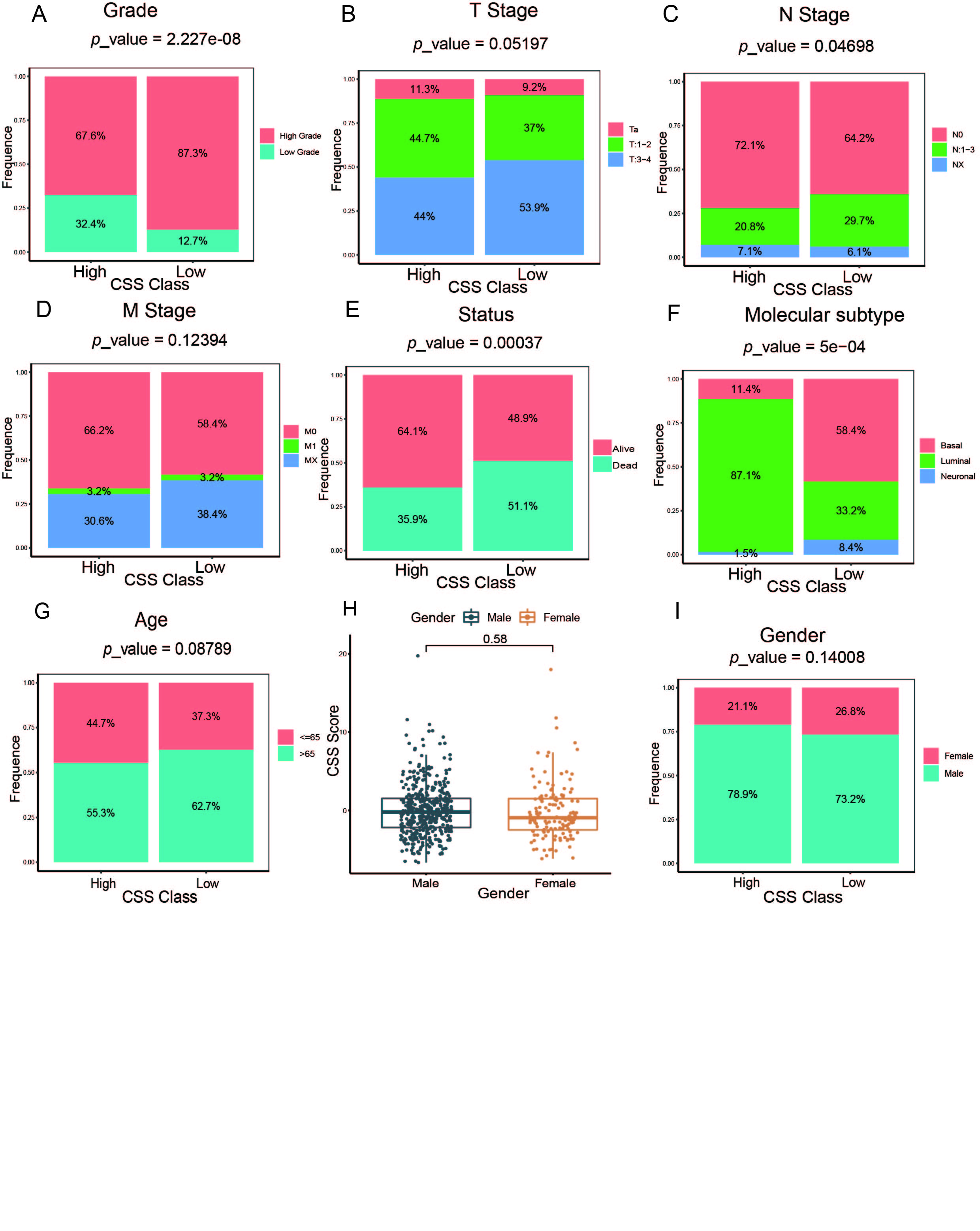

Supplement: Supplementary file 1 [file DataSheet_1.zip › Supplementary Figure S8.jpg]

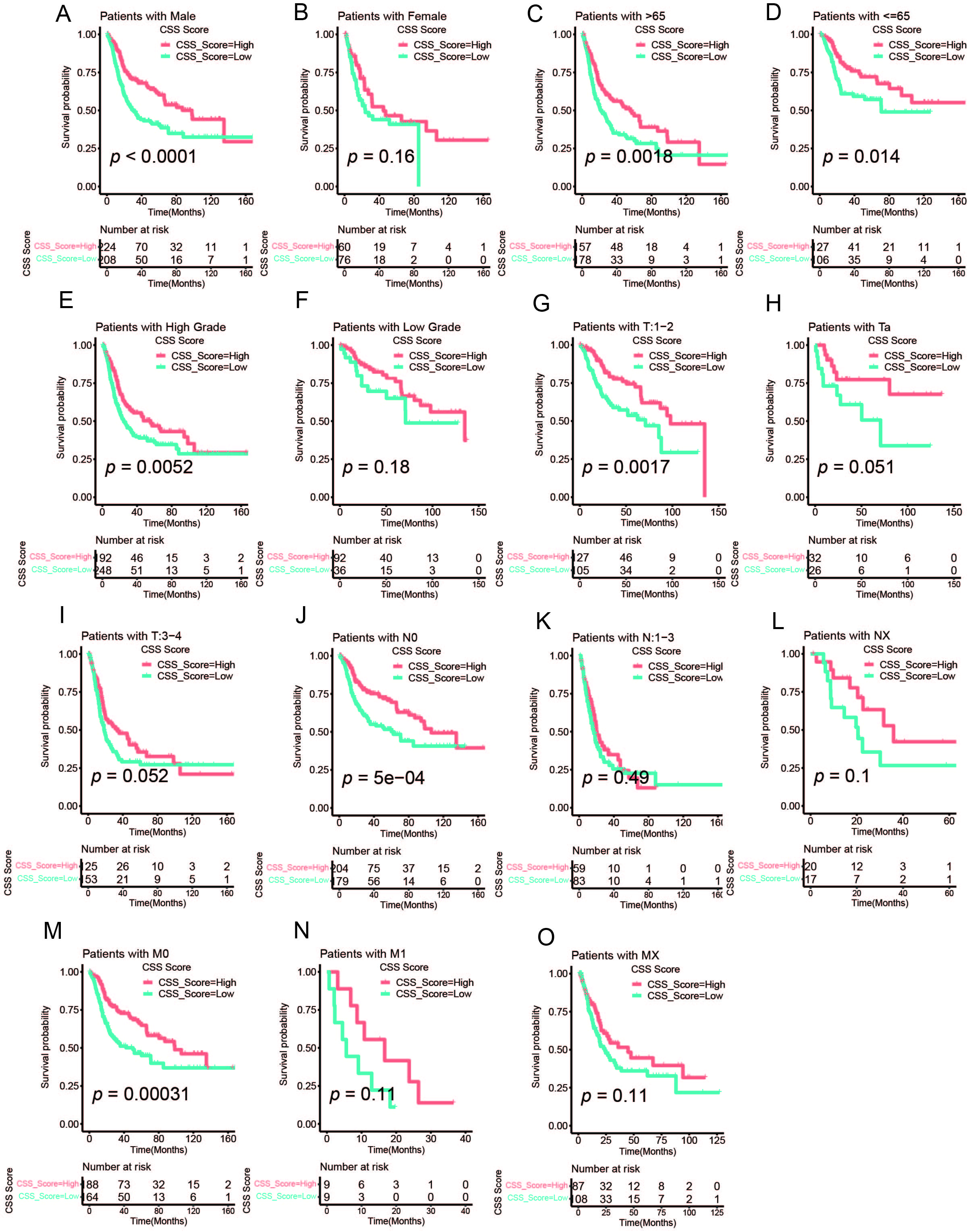

Supplement: Supplementary file 1 [file DataSheet_1.zip › Supplementary Figure S9.jpg]
